# Association of estimated glomerular filtration rate with stroke risk in middle-aged and older Chinese adults: an integrated analysis of national and hospital cohorts

**DOI:** 10.1265/ehpm.26-00008

**Published:** 2026-05-19

**Authors:** Jun Mao, Xue Wang, Xin Zhang, Mengyao Yu, Zhuowen Hu, Wuping Sun

**Affiliations:** Department of Clinical Laboratory, The Fifth Affiliated Hospital, Southern Medical University, Guangzhou, Guangdong 510910, China

**Keywords:** Stroke, eGFR, Global burden of disease, CHARLS, Kidney dysfunction

## Abstract

**Background:**

Stroke is a leading cause of death in China. While chronic kidney disease is a known stroke risk factor, quantitative evidence on the graded relationship between estimated glomerular filtration rate (eGFR) and stroke risk in the Chinese population remains limited.

**Methods:**

This study integrated individual-level data from the China Health and Retirement Longitudinal Study (CHARLS), including a prospective cohort (n = 7,786, follow-up from 2015 to 2020) and a cross-sectional cohort (n = 8,467, 2011 wave), alongside a hospital-based cross-sectional dataset (n = 1,649, including 839 patients with stroke). The association between eGFR (modeled as continuous and categorical variables) and stroke was assessed using multivariable Cox proportional hazards models. Additionally, Kaplan-Meier survival curves and log-rank tests were also performed. The dose-response relationship was examined using restricted cubic spline (RCS) analysis. Sensitivity analyses using multiple imputation were conducted.

**Results:**

Reduced eGFR was consistently associated with higher stroke risk across all datasets. In the fully adjusted prospective model, each 1 mL/min/1.73 m^2^ decrease in eGFR was associated with a 1.3% increase in stroke hazard (HR = 1.013, 95% CI: 1.006–1.020). Consistent associations were observed in the cross-sectional CHARLS cohort (OR = 1.020, 95% CI: 1.008–1.032) and the hospital cohort (OR = 1.026, 95% CI: 1.019–1.033). Kaplan-Meier curves showed significantly lower stroke-free survival in lower eGFR quartiles (log-rank P < 0.001). RCS analyses confirmed a linear inverse relationship in all datasets (P for overall < 0.001; P for nonlinear > 0.05). Subgroup analyses showed no significant effect modification by key demographics or comorbidities. Sensitivity analyses confirmed the robustness of the findings.

**Conclusion:**

Reduced eGFR is significantly and independently associated with increased stroke risk among Chinese adults aged ≥45 years, exhibiting a clear dose-response relationship. These findings support the potential utility of integrating renal function monitoring into stroke risk assessment and prevention strategies.

**Supplementary information:**

The online version contains supplementary material available at https://doi.org/10.1265/ehpm.26-00008.

## Introduction

Stroke is defined as a focal neurological deficit of acute onset resulting from the disruption of cerebral blood flow and constitutes a major global cause of death and long-term disability [1, 2], imposing an escalating public health and economic burden. Currently, stroke remains the second most common cause of death among non-communicable diseases globally [3]. Recent Global Burden of Disease (GBD) 2021 data indicate that China accounts for nearly 35.7% of global stroke-related deaths, with age-standardized incidence rates reaching 205 per 100,000, significantly exceeding the global average [4]. The economic burden in China is substantial. Reported direct costs in 2018 exceeded $58.6 billion, while indirect costs surpassed $166.5 billion [5]. Despite improved acute care, stroke recurrence remains clinically concerning (12–19.4% at 5 years) [6, 7]. Therefore, identifying additional independent risk factors is crucial for preventing stroke.

Estimated glomerular filtration rate (eGFR), a validated indicator of renal function, serves as an independent predictor of cerebrovascular events. Chronic kidney disease (CKD), defined as eGFR <60 mL/min/1.73 m^2^, represents a significant risk factor for stroke [8]. Mechanistically, CKD exacerbates endothelial dysfunction, accelerates atherosclerosis through the accumulation of uremic toxins (such as indoxyl sulfate), and promotes thrombosis through chronic inflammation, oxidative stress, and platelet hyperactivity [9–11].

Globally, multiple cohort studies and meta-analyses have established a graded, dose-response relationship between declining eGFR and increased stroke risk. A seminal meta-analysis encompassing over 285,000 participants from 33 studies reported a 43% increased stroke risk for eGFR <60 mL/min/1.73 m^2^, with a stronger association observed in Asian populations compared to Caucasians [12]. Subsequent studies have extended these findings to diverse population, in a German community cohort aged ≥70 years, eGFR 45–59 mL/min/1.73 m^2^ was found to be associated with a 2.2-fold increased risk of stroke, independent of albuminuria [13]; among Ghanaians with hypertension and diabetes, Sarfo et al. demonstrated a dose-dependent increase in stroke incidence across declining eGFR categories, with hazard ratios reaching 1.88 for eGFR 30–59 mL/min/1.73 m^2^ [14]; in addition, National Health and Nutrition Examination Survey (NHANES) in the US showed that eGFR <60 mL/min/1.73 m^2^ increased the odds of stroke by 56% compared with normal eGFR [15]. The National Health Insurance Service-National Sample Cohort (NHIS-NSC) in South Korea exhibited that reduced eGFR less than 45 mL/min/1.73 m^2^ was associated with an increased risk of ischemic stroke, especially in men [16]. Despite this accumulating international evidence, quantitative data on the eGFR-stroke dose-response relationship in the Chinese population remain limited. Furthermore, no study to date has integrated prospective community-based data with clinically validated hospital cohorts to assess generalizability and robustness simultaneously. This gap is particularly critical given China’s disproportionately high stroke burden and the rapidly aging population.

To address these gaps, the present study provides the first integrated analysis of the eGFR-stroke association in Chinese adults aged ≥45 years using a dual-design approach. We harmonize data from the nationally representative China Health and Retirement Longitudinal Study (CHARLS), including both prospective (2015–2020) and cross-sectional (2011) cohorts, with an independent hospital-based validation cohort from a tertiary stroke center. This design allows us to quantify the dose-response relationship using both categorical and continuous eGFR modeling and generate population-relevant risk estimates to inform targeted prevention strategies in China’s aging society.

## Methods

### Data sources and study populations

#### China Health and Retirement Longitudinal Study (CHARLS) database

CHARLS is a longitudinal follow-up study of middle-aged and elderly individuals aged ≥45 years and their spouses in China, coordinated by Peking University. The study utilizes a multi-stage, stratified probability sampling design to ensure national representativeness of community-dwelling residents aged ≥45 years (excluding Tibet). The baseline survey encompassed 450 villages across approximately 150 counties, recruiting 17,708 respondents from 10,257 households. Biennial follow-ups collect detailed information on respondents’ demographic characteristics, health status, family structure, employment status, and retirement patterns [17]. For this study, cross-sectional analyses utilized data from the 2011 CHARLS wave, while prospective cohort analyses utilized data from 2015 to 2020 (wave 3 to wave 5). Inclusion criteria were as follows: (1) age ≥45 years, this threshold was chosen because it aligns with the sampling frame of the CHARLS survey, captures the age range in which stroke incidence in China begins to rise substantially; (2) availability of complete data for body mass index (BMI), serum creatinine, gender, age, and stroke status; (3) provision of a fasting blood sample; and (4) BMI 10–100 kg/m^2^. Exclusion criteria comprised: (1) failure to meet any inclusion criterion; (2) loss to follow-up in the 2020 wave; (3) a pre-existing stroke diagnosis at baseline (Wave 3, 2015). Thus, for all participants in the prospective analysis, the baseline eGFR measurement was obtained before the occurrence of any incident stroke event identified during follow-up. Ultimately, the final analytical samples consisted of 8,467 participants for the cross-sectional analysis and 7,786 for the prospective cohort analysis. The study received approval from Peking University’s Biomedical Ethics Committee (IRB00001052-11015), and all participants provided written informed consent.

#### Hospital-based cross-sectional dataset

To examine the consistency of the eGFR-stroke association in a clinical inpatient population, we retrospectively collected data from patients hospitalized in the Department of Neurology (Stroke Center) at the Fifth Affiliated Hospital of Southern Medical University between January 2020 and December 2024. The inclusion criteria comprised: (1) age ≥45 years; (2) serum creatinine was measured within 24 hours of admission; and (3) had a clear primary diagnosis of discharge. Patients were divided into two groups based on diagnosis: stroke group (patients with acute stroke confirmed by imaging) and non-stroke group (patients hospitalized for non-cerebrovascular neurological diseases and no history of stroke). Stroke diagnosis required confirmation by MRI/CT according to WHO criteria. Exclusion criteria comprised: (1) missing key data (age, gender, creatinine, diagnosis); (2) Patients with end-stage renal disease or dialysis; (3) Acute kidney injury before admission; (4) non-stroke diseases such as brain trauma, brain tumor, intracranial infection, etc.; (5) Hospitalization less than 24 hours. The final cohort comprised 1,649 patients: 839 stroke patients and 810 non-stroke controls. As this study analyzed de-identified aggregate data, the Ethics Review Committee waived the requirement for informed consent (2025-JYYXK-K-004). Since this study is not a clinical trial, Clinical trial number: not applicable.

### Variable definitions

Serum creatinine (SCr) measurements for 2011 and 2015 CHARLS waves were performed by the China CDC using the rate-blanked compensated kinetic Jaffe method; this study applied the coefficient-modified CKD-EPI equation from Japanese [18] to calculate eGFR. Participants with eGFR <60 mL/min/1.73 m^2^ were classified with kidney dysfunction. For the cross-sectional analysis, eGFR stages were defined according to the Kidney Disease: Improving Global Outcomes (KDIGO) guidelines [19]: G1 ≥90, G2 60–89, G3a 45–59, G3b 30–44, G4 15–29, and G5 <15 mL/min/1.73 m^2^; For prospective cohort analysis, participants were categorized into quartiles based on their eGFR values (Q1–Q4).

Covariates included: age, gender (male/female), marriage (married/other), residence (rural/urban), education (primary school or lower/middle school/high school or above), BMI, smoking history (yes/no), drinking history (yes/no), and self-reported histories of kidney disease, diabetes, hypertension, heart disease, and dyslipidemia (yes/no); fasting blood glucose (FBG), low-density lipoprotein cholesterol (LDL-c).

Stroke served as the primary outcome, ascertained through participant self-report. Trained interviewers administered the question: “Has a physician ever diagnosed you with stroke?” Affirmative responses were recorded as stroke cases. Stroke subtype information was not available in CHARLS. In the hospital-based cohort, both ischemic and hemorrhagic strokes were recorded. For primary analyses across all cohorts, total stroke was used as the outcome. Subtype-specific analyses were additionally conducted in the hospital cohort.

### Statistical analysis

For the CHARLS database and hospital validation cohort, categorical variables are presented as frequencies (%), and continuous variables as means ± standard deviations, respectively. Between-group differences in categorical variables were assessed using the χ^2^ test, while continuous variables were compared via the Kruskal-Wallis H test. For multicollinearity testing, we used the Generalized Variance Inflation Factor (GVIF) to evaluate each variable, and the GVIF^1/2Df^ of all variables in our study was less than 2 [20] (Table S1). For cross-sectional data, binary logistic regression was used to examine associations between eGFR and stroke. For the prospective CHARLS cohort, we employed Cox proportional hazards models to estimate the association between eGFR and incident stroke. Stroke events were self-reported retrospectively at each wave. The CHARLS survey does not record precise stroke onset dates. In the 2018 wave, a minority of participants reported the year of diagnosis; no month or exact date was available. In the 2020 wave, no timing information was provided for any incident case. To enable Cox regression while minimizing bias, we adopted a midpoint imputation strategy consistent with prior studies using interval-censored survey data. For events first reported in 2018 with a recorded year, we used that year. For events first reported in 2018 without a recorded year, we assigned the midpoint of the 2015–2018 interval (2017). For all events first reported in 2020, we assigned the midpoint of the 2018–2020 interval (2019). Three hierarchical models were specified: Model 1: unadjusted; Model 2: adjusted for age, gender, marriage, residence, education and BMI; Model 3: Model 2 variables plus smoking, drinking, kidney disease, diabetes, heart disease, dyslipidemia, FBG and LDL-c; The proportional hazards assumption was tested using Schoenfeld residuals; no evidence of violation was found (global P > 0.05). Survival curves for eGFR quartiles were generated using the Kaplan-Meier method and compared using the log-rank test. Additionally, the linearity and dose-response relationship between eGFR and stroke was assessed using restricted cubic spline (RCS) with four knots at the 5th, 35th, 65th and 95th percentiles of the eGFR. Finally, subgroup analyses were conducted with stratification by age (<60 vs. ≥60 years), gender, marriage, residence, drinking, education level, kidney disease, diabetes, hypertension, heart disease, and dyslipidemia. Multiplicative interaction terms were tested.

Data cleaning and preprocessing of the CHARLS raw data were conducted using Stata 18. All statistical analyses were conducted in R (v4.3.1), with two-tailed P < 0.05 indicating statistical significance.

### Sensitivity analysis

To assess the robustness of the primary findings and address potential bias due to missing covariate data, we performed multiple imputation analyses. Multiple imputation by chained equations (MICE) was employed to generate five complete datasets, incorporating all variables used in the primary regression models (Model 3), including the outcome variable (stroke status) to ensure proper imputation. The imputation model specifications, including the list of imputed variables, imputation methods, and convergence diagnostics, are detailed in Additional file 1. The results from the multivariable Cox regression models were applied to each imputed dataset and subsequently pooled using Rubin’s rules to obtain combined hazard ratios (HRs) and 95% confidence intervals (CIs). The findings from these sensitivity analyses were compared with those from the complete-case analysis to evaluate consistency.

## Results

### Baseline characteristics of the study population

**Fig. 1:**
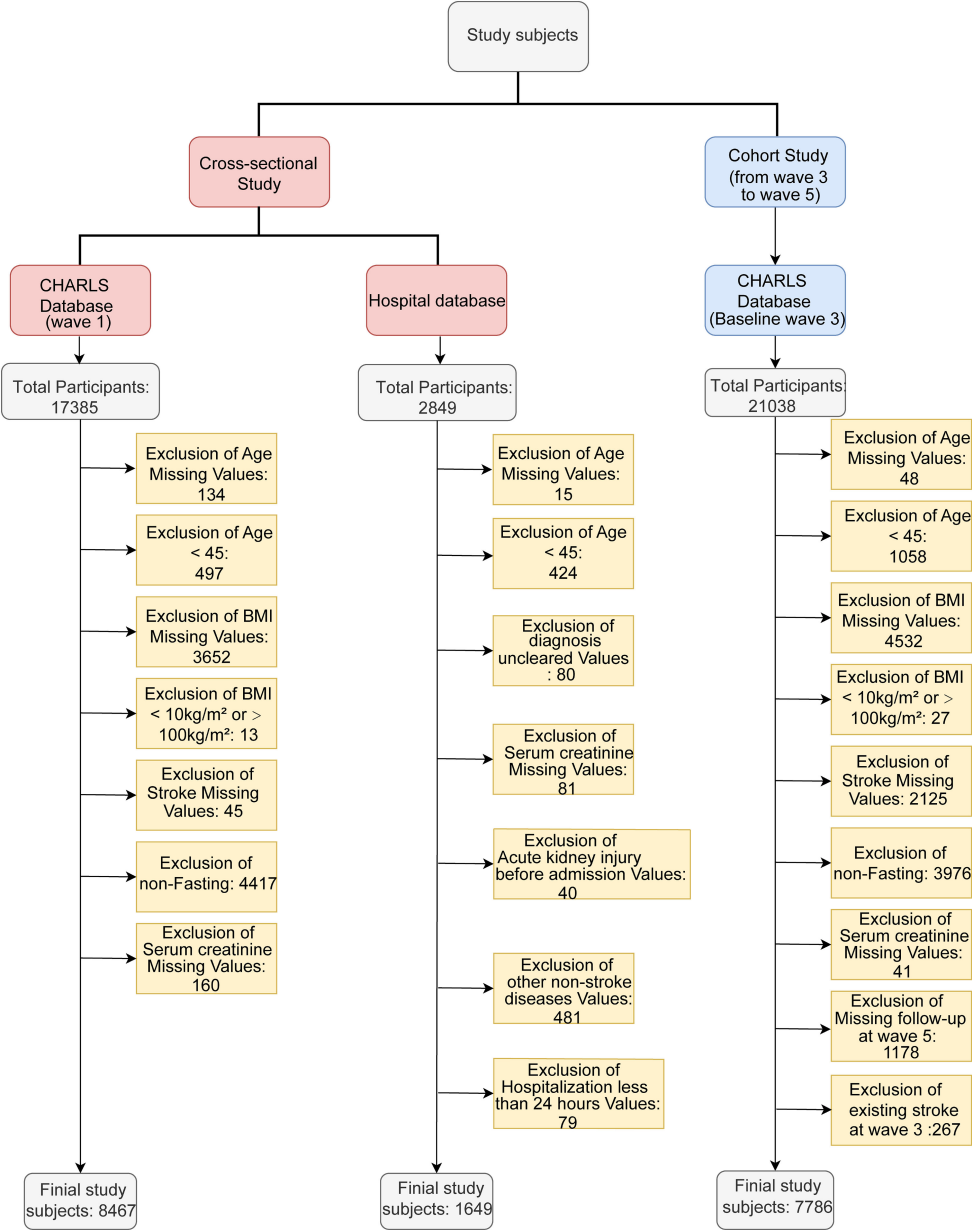
Flowchart of CHARLS and hospital cohort.

As seen in Fig. 1, the prospective cohort analysis included a baseline sample of 7,786 participants (3,441 males [44.2%] and 4,345 females [55.8%]), with a mean age of 61.1 ± 8.9 years. Participants were stratified into four groups according to eGFR quartiles (Q1–Q4) (Table 1). Compared with the Q4 group (highest eGFR), participants in lower eGFR quartiles (Q1–Q3) were older, had a lower prevalence of being married, a lower proportion of rural residence, and lower educational attainment (all P < 0.001). Additionally, they had elevated FBG and LDL-c levels, as well as a higher prevalence of kidney disease, hypertension, heart disease, and dyslipidemia (all P < 0.05). In the cross-sectional analysis, a similar pattern of characteristics was observed in the 2011 CHARLS dataset and the hospital-based cross-sectional dataset. Moreover, participants with more advanced renal impairment (stages G3a–G5) had a higher prevalence of stroke (P < 0.001; Tables S2 and S3).

**Table 1:** Baseline characteristics of individuals classified by categories of the eGFR.

**Characteristics**	**Overall**	**Categories of eGFR**				**P value**

		**Quartile 1**	**Quartile 2**	**Quartile 3**	**Quartile 4**	
**n**	7786	1948	1945	1946	1947	
**Gender (Male) (%)**	3441 (44.2)	953 (48.9)	901 (46.3)	867 (44.6)	720 (37.0)	<0.001
**age (mean (SD)), years**	61.1 (8.9)	66.7 (9.1)	64.6 (8.1)	59.8 (5.9)	53.3 (5.1)	<0.001
**Marriage (Married) (%)**	6799 (87.3)	1563 (80.2)	1639 (84.3)	1763 (90.6)	1834 (94.2)	<0.001
**Residence (Rural) (%)**	5057 (64.9)	1185 (60.8)	1267 (65.1)	1289 (66.2)	1316 (67.6)	<0.001
**education (%)**						<0.001
**Primary school or lower**	5258 (67.5)	1409 (72.3)	1370 (70.4)	1318 (67.7)	1161 (59.6)	
**Middle school**	1682 (21.6)	341 (17.5)	380 (19.5)	407 (20.9)	554 (28.5)	
**High school or above**	846 (10.9)	198 (10.2)	195 (10.0)	221 (11.4)	232 (11.9)	
**BMI (mean (SD)), kg/m^2^**	24.0 (4.0)	23.8 (3.9)	23.7 (4.0)	24.1 (4.3)	24.6 (4.0)	<0.001
**Drinking (Yes) (%)**	3540 (45.5)	902 (46.4)	920 (47.4)	874 (44.9)	844 (43.4)	0.068
**Smoking (Yes) (%)**	3232 (41.5)	873 (44.8)	838 (43.1)	841 (43.2)	680 (34.9)	<0.001
**Kidney disease (Yes) (%)**	703 (9.1)	208 (10.8)	165 (8.5)	193 (10.0)	137 (7.1)	<0.001
**Diabetes (Yes) (%)**	1430 (18.6)	368 (19.1)	350 (18.2)	341 (17.7)	371 (19.3)	0.567
**Hypertension (Yes) (%)**	3963 (51.3)	1153 (59.5)	1031 (53.4)	955 (49.5)	824 (42.7)	<0.001
**Heart disease (Yes) (%)**	1320 (17.2)	398 (20.7)	367 (19.2)	321 (16.7)	234 (12.2)	<0.001
**Dyslipidemia (Yes) (%)**	1455 (19.4)	403 (21.5)	362 (19.4)	373 (19.7)	317 (17.0)	0.006
**FBG (mean (SD)), mg/dL**	100.3 (29.0)	99.8 (25.4)	98.5 (23.3)	99.8 (26.6)	103.1 (38.3)	<0.001
**LDL-c (mean (SD)),** **mg/dL**	103.1 (29.1)	105.9 (29.6)	105.5 (29.6)	101.9 (28.0)	99.2 (28.5)	<0.001
**CREA (mean (SD)),** **mg/dL**	0.8 (0.3)	1.0 (0.4)	0.8 (0.1)	0.7 (0.1)	0.6 (0.1)	<0.001
**eGFR (mean (SD)),** **mL/min/1.73 m^2^**	73.3 (12.5)	56.4 (11.1)	72.2 (2.4)	78.7 (1.6)	86.0 (3.6)	<0.001

BMI, body mass index; FBG, fasting blood glucose; LDL-c, low-density lipoprotein cholesterol; CREA, serum creatinine; eGFR, estimated glomerular filtration rate.

### Analysis of the association between eGFR and stroke

Univariate and multivariate logistic regression analyses were performed to elucidate the eGFR-stroke relationship. These analyses revealed that eGFR, when modeled as a continuous variable, was significantly associated with stroke risk in the unadjusted, partially adjusted, and fully adjusted models (Table 2). In the fully adjusted model, each 1 mL/min/1.73 m^2^ decrease in eGFR was associated with a 1.3% higher hazard of incident stroke (HR = 1.013, 95% CI: 1.006–1.020). Consistently, when analyzed as a categorical variable, the fully adjusted models indicated that the lower eGFR quartiles (Q1–Q3) were associated with significantly higher hazard of incident stroke compared to the Q4 reference group (highest eGFR) in the prospective cohort (Q1: HR = 2.032, 95% CI: 1.500–2.752; Q2: HR = 1.430, 95% CI: 1.053–1.943; Q3: HR = 1.361, 95% CI: 1.017–1.821). Similarly, analyses of the cross-sectional data also demonstrated a significant association between eGFR and stroke. The fully adjusted models for the cross-sectional analyses indicated that each 1 mL/min/1.73 m^2^ decrease in eGFR was associated with a 2.0% increase in stroke odds in the 2011 CHARLS dataset (OR = 1.020, 95% CI: 1.008–1.032), and a 2.6% increase in the hospital-based cohort (OR = 1.026, 95% CI: 1.019–1.033). Accordingly, when categorized by KDIGO stage, the G3a–G5 groups demonstrated significantly higher odds of stroke compared to the G1 stage (all P for trend < 0.05; Tables S4 and S5). When stroke was classified by subtype in the hospital-based cohort, the association between reduced eGFR and stroke remained significant for both ischemic and hemorrhagic stroke. In fully adjusted models, each 1 mL/min/1.73 m^2^ decrease in eGFR was associated with a 2.5% increase in odds of ischemic stroke (OR = 1.025, 95% CI: 1.016–1.034) and a 2.7% increase in odds of hemorrhagic stroke (OR = 1.027, 95% CI: 1.018–1.035). Categorical analyses by KDIGO stage similarly demonstrated progressively higher odds of both stroke subtypes with declining eGFR (all P for trend < 0.001, Tables S6 and S7).

**Table 2:** Association between the eGFR and Stroke.

**eGFR**	**Categories**				**P for trend**	**Continuous** **Per 1 mL/min/1.73** **m^2^ decrease**

	**Quartile 1**	**Quartile 2**	**Quartile 3**	**Quartile 4**		
**Median**	59.72	72.39	78.66	85.19		
**Cases, n (%)**	208 (11.8)	144 (8.1)	121 (6.9)	81 (4.6)		
**Model 1** **HR (95% CI)**	2.712 (2.112–3.482)	1.813 (1.390–2.364)	1.606 (1.224–2.106)	Ref	<0.001	1.021 (1.015–1.026)
**Model 2** **HR (95% CI)**	2.123 (1.588–2.850)	1.485 (1.107–1.993)	1.437 (1.085–1.902)	Ref	<0.001	1.014 (1.007–1.020)
**Model 3** **HR (95% CI)**	2.032 (1.500–2.752)	1.430 (1.053–1.943)	1.361 (1.017–1.821)	Ref	<0.001	1.013 (1.006–1.020)

Model 1: adjusted for no variables;

Model 2: adjusted for age, gender, marriage, residence, education, and BMI.

Model 3: adjusted for variables included in Model 2 and drinking history, smoking history, kidney disease, diabetes, hypertension, heart disease, dyslipidemia, FBG, and LDL-c.

eGFR, estimated glomerular filtration rate; HR, hazard ratio; CI, confidence interval.

Figure S1 presents the Kaplan-Meier cumulative incidence curves for stroke according to eGFR quartiles. Participants in the lowest eGFR quartile (Q1) exhibited the lowest stroke-free survival probability, whereas those in the highest quartile (Q4) had the highest survival probability (log-rank P < 0.001).

**Fig. 2:**
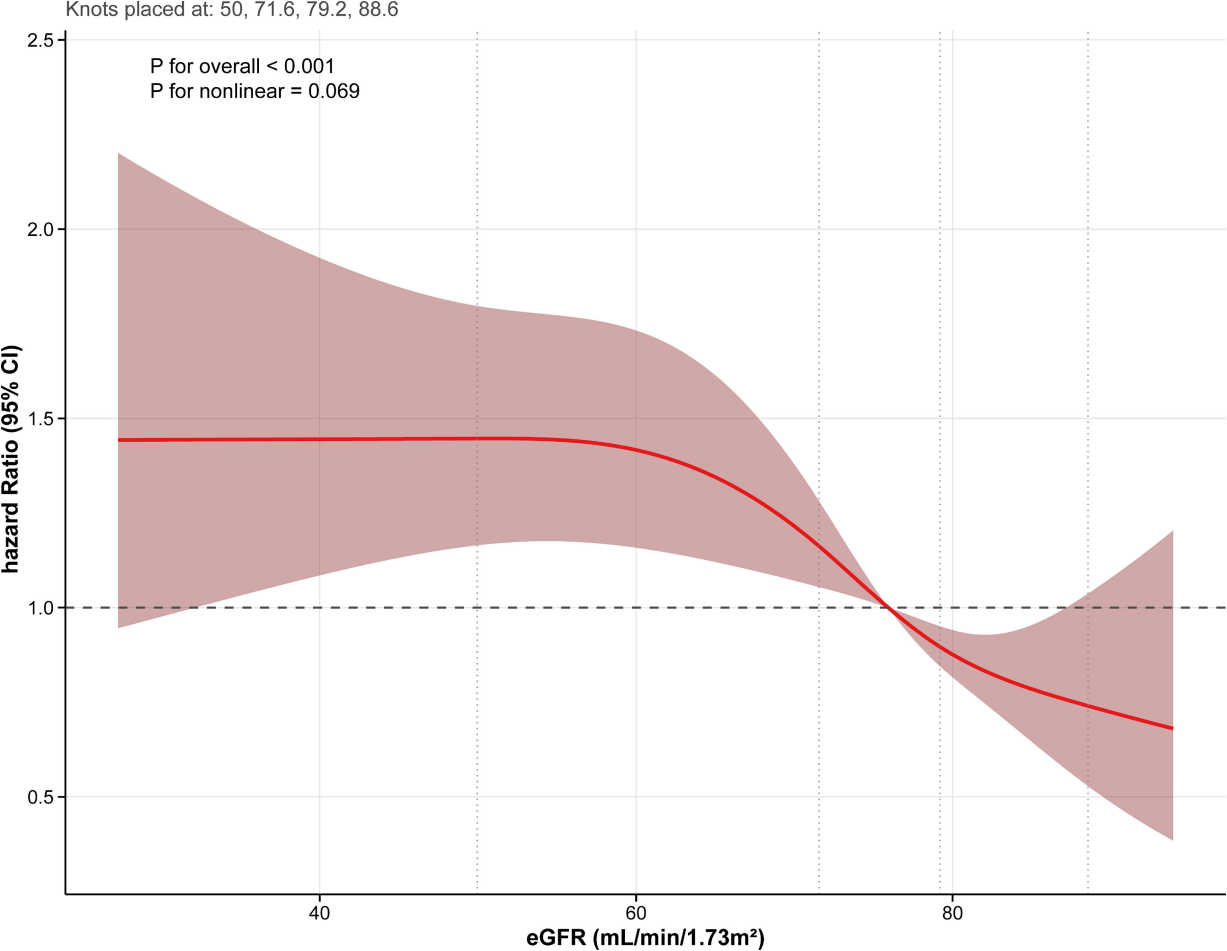
Association between the eGFR and stroke. RCS model in CHARLS was adjusted for age, gender, marriage, residence, BMI, smoking history, drinking history, education level, hypertension, dyslipidemia, diabetes, kidney disease, heart disease, FBG, and LDL-c.

Restricted cubic spline analysis revealed a linear inverse relationship between eGFR and stroke incidence (P for overall < 0.001; P for nonlinear = 0.069; Fig. 2). This linear inverse relationship was consistent in both the hospital-based cohort (P for overall < 0.001; P for nonlinear = 0.762; Fig. S2) and the 2011 CHARLS cohort (P for overall < 0.001; P for nonlinear = 0.348; Fig. S3). For subtypes in the hospital-based cohort, restricted cubic spline analyses confirmed linear inverse relationships for both subtypes (P for overall < 0.001; P for nonlinear > 0.05; Figs. S4–S5). These results indicate that the eGFR-stroke association is consistent across major stroke types.

### Subgroup analysis

**Fig. 3:**
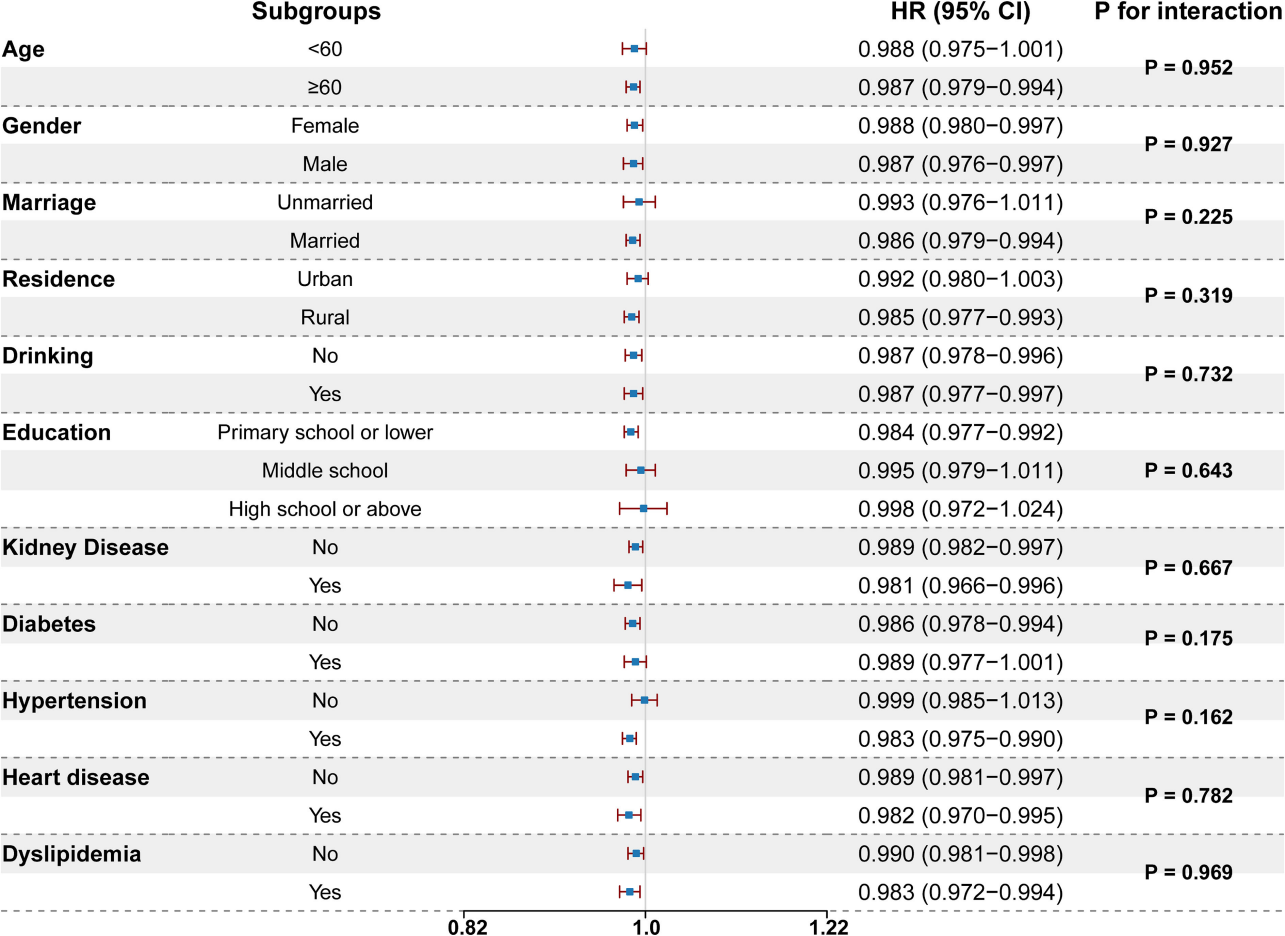
Subgroup analyses of the association between the eGFR and stroke. The model was adjusted for age, gender, marriage, residence, education level, BMI, smoking history, drinking history, education level, hypertension, dyslipidemia, diabetes, liver disease, kidney disease, FBG, and LDL-c. BMI, body mass index; FBG, fasting blood glucose; LDL-c, low-density lipoprotein cholesterol; HR, hazard ratio; CI, confidence interval; eGFR, estimated glomerular filtration rate.

We conducted subgroup analyses to assess the consistency of the association between eGFR and stroke across key demographic and clinical strata. These analyses, stratified by age, gender, marital status, residence, drinking history, education attainment, kidney disease, diabetes, hypertension, heart disease, and dyslipidemia, revealed that none of the subgroups changed the associations between reduced eGFR and increased stroke incidence (all P for interaction > 0.05; Fig. 3, S6).

### Sensitivity analysis

The results from the fully adjusted models following multiple imputation were consistent with the primary analysis: (Q1: HR = 1.988, 95% CI: 1.487–2.659; Q2: HR = 1.476, 95% CI: 1.102–1.978; Q3: HR = 1.335, 95% CI: 1.008–1.767; per 1 mL/min/1.73 m^2^ decrease: HR = 1.012, 95% CI: 1.006–1.019; Table S8). Similarly, in the CHARLS 2011 dataset, the results obtained after multiple imputation aligned with those of the primary analysis. The analysis demonstrated a 2.1% increase in stroke risk per 1 mL/min/1.73 m^2^ decrease in eGFR (OR = 1.021; 95% CI: 1.012–1.030). Furthermore, the analysis by KDIGO stage also confirmed that participants with more advanced renal impairment (G3a–G5) had significantly higher odds of stroke relative to the G1 reference group (all P for trend < 0.05; Table S9).

## Discussion

This integrated study provides robust evidence for a significant, independent, and linear inverse association between reduced eGFR and increased stroke risk among Chinese adults aged ≥45 years. By harmonizing nationally representative prospective data with a hospital-based validation cohort, our findings not only corroborate the well-established link between CKD and stroke documented in global meta-analyses [21], but also significantly extend this knowledge by providing the first detailed dose-response characterization and cross-setting validation specifically for the high-risk Chinese population.

Our results are quantitatively consistent with prior large-scale evidence. The observed 1.3% increase in stroke hazard per 1 mL/min/1.73 m^2^ decrease in eGFR in our prospective cohort is in line with a landmark meta-analysis [21], which reported a 7% increase in risk per 10 mL/min/1.73 m^2^ decrement. Furthermore, our restricted cubic spline analyses confirmed a linear dose-response relationship across all three datasets, reinforcing the concept that stroke risk increases progressively with declining kidney function. This moves beyond earlier studies that were often limited to categorical comparisons of CKD stages [8, 21] and provides a more nuanced risk gradient for clinical application.

The consistency of our findings with recent international cohort studies further strengthens our validity, while the comparisons also illuminate important context-specific nuances. For instance, in the Berlin Initiative Study of adults aged ≥70 years, an eGFR of 45–59 mL/min/1.73 m^2^ was strongly associated with stroke (HR = 2.23) but not with myocardial infarction, suggesting a potentially unique cerebro-renal link in older adults [13]. Our study extends this observation to a younger (≥45 years) and ethnically distinct Chinese cohort, demonstrating that the eGFR-stroke association is already evident in middle age and is robust across different demographic and clinical subgroups. Similarly, Sarfo et al., prospectively demonstrated a dose-response relationship between eGFR and incident stroke in Ghanaians with hypertension and diabetes, with a particularly pronounced effect in hypertensive patients (HR = 3.69 for eGFR <60 mL/min/1.73 m^2^) [14]. While our study also found a significant association in the general population after full adjustment, the effect size was more modest (HR = 2.032 for the lowest eGFR quartile). This discrepancy may reflect differences in baseline risk profiles, as our community-based sample from CHARLS included individuals with a lower prevalence of severe comorbidities compared to the hospital-based, high-risk African cohort. This comparison underscores the importance of our integrated design, which combines a generalizable community cohort with a high-specificity hospital cohort, providing a more complete analysis than either setting alone.

Our findings from cross-sectional analyses within CHARLS and the hospital cohort further reinforce the main conclusion. Xiang et al., using NHANES (2007–2018) cross-sectional data, also reported a significant negative linear correlation between eGFR and prevalent stroke in CKD patients [22]. However, our study advances this cross-sectional evidence by demonstrating that the association holds in a nationally representative sample of community-dwelling adults, not just in those with established CKD. Moreover, the consistency of the linear relationship observed in both our community and clinical samples with the prospective RCS analysis provides convincing evidence that mitigates the inherent limitations of any single study design.

While our study did not measure specific pathological mediators, the observed association is biologically plausible and consistent with established studies on the multifaceted pathophysiology of CKD. This pathophysiology likely involves synergistic effects of traditional and non-traditional mechanisms. Traditional stroke risk factors, like hypertension, diabetes, carotid artery disease, cardiovascular disease, obesity, and dyslipidemia, are concomitantly prevalent and exacerbated in CKD patients [8, 23, 24]. Concurrently, CKD-specific non-traditional mechanisms, including atherosclerosis acceleration through uremic toxin accumulation [9, 10, 25], thrombotic enhancement [11], chronic inflammation [26], oxidative stress [27], and impaired endothelial function [28], are well-documented. It is plausible that these intertwined processes collectively foster a pro-thrombotic and pro-atherogenic state in patients with declining renal function, thereby increasing stroke susceptibility.

The strengths of this study lie in its multidimensional design, harmonizing prospective and cross-sectional data from a large, nationally representative community cohort with a rigorously matched hospital-based validation cohort. This approach strengthens internal validity and enhances the clinical relevance of our findings. The utilization of CHARLS facilitated robust adjustment for a wide array of confounders, while sensitivity analyses employing multiple imputation confirmed the robustness of the core association. Comprehensive subgroup analyses further bolstered the credibility of our assessment regarding the correlation between the eGFR and stroke. However, several limitations warrant consideration. First and foremost, the issue of reverse causation must be carefully considered. Two of our three datasets are cross-sectional, which inherently precludes causal inference. Although our primary analysis utilized prospective data from CHARLS (2015–2020), with baseline eGFR measured prior to self-reported incident stroke, reverse causality remains a plausible alternative explanation. Subclinical cerebrovascular diseases may precede and contribute to both eGFR decline and eventual clinical stroke; the observed association could partly reflect unmeasured confounding rather than a direct causal effect. Future studies using causal inference methods are needed to confirm directionality. Second, the ascertainment of stroke outcomes differed substantially between the CHARLS and hospital-based cohorts, a methodological heterogeneity that warrants careful consideration. In the CHARLS cohorts, stroke was defined by self-reported physician diagnosis, a method that is practical for large-scale population surveys but is susceptible to recall bias and under-ascertainment of mild, transient, or silent cerebrovascular events. This misclassification is likely non-differential with respect to eGFR, particularly after comprehensive adjustment for sociodemographic and clinical covariates; non-differential outcome misclassification of a binary endpoint invariably biases effect estimates toward the null. Consequently, the true association between reduced eGFR and stroke risk in the general Chinese population may be even stronger than the odds ratios and hazard ratios estimated from the CHARLS data. In contrast, the hospital cohort used imaging-confirmed diagnoses, ensuring high specificity but introducing selection bias. The stroke group comprised patients hospitalized with moderate-to-severe acute strokes, potentially enriching the case group for low eGFR. While the control group consisted of patients admitted for other non-cerebrovascular neurological disorders, not healthy community dwellers. The selection tends to inflate the observed eGFR-stroke association compared with the true effect in the general population and limits the generalizability of absolute effect estimates. Despite the distinct and oppositely directed biases operating in the two settings, both cohorts consistently demonstrated a significant, graded, inverse association between eGFR and stroke odds. This convergence of evidence across settings with differing limitations strengthens the credibility of our core finding. However, the heterogeneity in case ascertainment and control selection precludes direct quantitative comparison of effect sizes between the CHARLS and hospital cohorts. Instead, the primary value of the hospital cohort lies in its independent validation of the association pattern within a clinically relevant inpatient context. Future prospective studies employing standardized, imaging-confirmed stroke ascertainment in community-based populations would be valuable to obtain unbiased effect estimates. Third, although we adopted Cox proportional hazards models for the prospective CHARLS cohort, the imprecision of stroke onset timing remains a limitation. The majority of events could only be localized to a two-year survey interval, necessitating midpoint imputation. While this method is widely used for interval-censored data and our sensitivity analyses confirmed robustness, it may still introduce some degree of nondifferential misclassification, potentially biasing hazard ratios toward the null. Future studies with active surveillance and adjudicated event dates are warranted to obtain more precise effect estimates. Fourth, kidney dysfunction was defined solely by eGFR without urinary albumin measurements, potentially leading to under-identification of early-stage CKD. Fifth, unmeasured confounders (e.g., medication adherence, dietary patterns, genetic susceptibility) may persist despite extensive covariate adjustments. Moreover, the potential mediating role of some adjusted confounders in the eGFR-stroke relationship was not explored. Sixth, our study was restricted to Chinese adults aged ≥45 years, limiting generalizability to younger populations or other ethnicities. Therefore, caution is warranted when extrapolating our results to younger age groups. Seventh, although our primary analysis combined all stroke types due to data limitations in CHARLS, the additional subtype-specific analyses in the hospital cohort demonstrated consistent associations for both ischemic and hemorrhagic stroke, suggesting that the observed eGFR-stroke relationship is not driven by a single pathological mechanism. Nevertheless, future studies with comprehensive neuroimaging in community settings are needed to confirm these findings and explore potential differences in effect size. Finally, the sample size in certain advanced eGFR categories was limited, resulting in wide confidence intervals; thus, larger studies are needed for more precise estimates in these high-risk subgroups.

## Conclusion

In conclusion, prospective data from a national cohort suggest that reduced eGFR is associated with an increased risk of incident stroke. This association is strongly and consistently observed in cross-sectional analyses of both community-dwelling and clinical inpatient populations, indicating its broad relevance. These findings underscore the importance of considering renal function in comprehensive assessments of vascular health. Integrating routine eGFR assessment into existing stroke risk prediction models and developing targeted management strategies for individuals with impaired renal function, particularly those in advanced CKD stages, represent promising avenues for enhancing primary stroke prevention in China’s aging population. Future interventional studies are warranted to evaluate the efficacy and cost-effectiveness of such integrated approaches.

## Supplementary information

**Additional file 1:** Imputation model specifications and convergence diagnostics.

**Additional file 2:** **Table S1:** Collinearity statistics for all cohorts.

**Additional file 3:** **Table S2:** Baseline characteristics by eGFR category (CHARLS 2011 wave).

**Additional file 4:** **Table S3:** Baseline characteristics by eGFR category (hospital cohort).

**Additional file 5:** **Table S4:** Association between eGFR and stroke (CHARLS 2011 wave).

**Additional file 6:** **Table S5:** Association between eGFR and stroke (hospital cohort).

**Additional file 7:** **Table S6:** Association between eGFR and ischemic stroke in the hospital cohort.

**Additional file 8:** **Table S7:** Association between eGFR and hemorrhagic stroke in the hospital cohort.

**Additional file 9:** **Table S8:** Association between eGFR and stroke after multiple imputation (prospective cohort).

**Additional file 10:** **Table S9:** Association between eGFR and stroke after multiple imputation (CHARLS 2011 wave).

**Additional file 11:** **Figure S1:** Kaplan-Meier survival curve for new-onset stroke of CHARLS participants by eGFR quartile.

**Additional file 12:** **Figure S2:** Dose-response relationship between eGFR and stroke (hospital cohort, RCS analysis).

**Additional file 13:** **Figure S3:** Dose-response relationship between eGFR and stroke (CHARLS 2011 wave, RCS analysis).

**Additional file 14:** **Figure S4:** Dose-response relationship between eGFR and ischemic stroke in the hospital cohort (RCS analysis).

**Additional file 15:** **Figure S5:** Dose-response relationship between eGFR and hemorrhagic stroke in the hospital cohort (RCS analysis).

**Additional file 16:** **Figure S6:** Subgroup analysis of the eGFR-stroke association (CHARLS 2011 wave).

## Declarations

### Availability of data and materials

The data reported in this paper is available from http://charls.pku.edu.cn/.
