# Supplementary material for: Association of estimated glomerular filtration rate with stroke risk in middle-aged and older Chinese adults: an integrated analysis of national and hospital cohorts
Source: Environ Health Prev Med. 2026 May 19;31:33. doi: 10.1265/ehpm.26-00008 (PMC13222745; doi:10.1265/ehpm.26-00008)
Supplement: Supplementary file 1 — Additional file 1: Imputation model specifications and convergence diagnostics. [file ehpm-31-033-s001.docx]

Prospective cohort (2015-2020) and 2011 cohort

Imputation variables and methods

| **CREA** | "pmm" |
| --- | --- |
| **Age** | "pmm" |
| **BMI** | "pmm" |
| **LDL-c** | "pmm" |
| **FBG** | "pmm" |
| **education** | "polyreg" |
| **Gender** | "logreg" |
| **Smoking** | "logreg" |
| **Marriage** | "logreg" |
| **Residence** | "logreg" |
| **Drinking** | "logreg" |
| **Kidney disease** | "logreg" |
| **Diabetes** | "logreg" |
| **Hypertension** | "logreg" |
| **Heart disease** | "logreg" |
| **Dyslipidemia** | "logreg" |

BMI, body mass index; FBG, fasting blood glucose; LDL-c, low-density lipoprotein cholesterol; CREA, serum creatinine.

Number of imputations: 5

maxit = 10


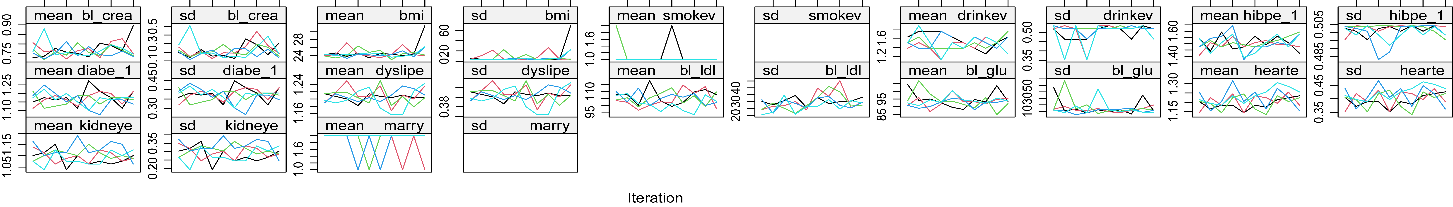


prospective cohort (2015-2020)


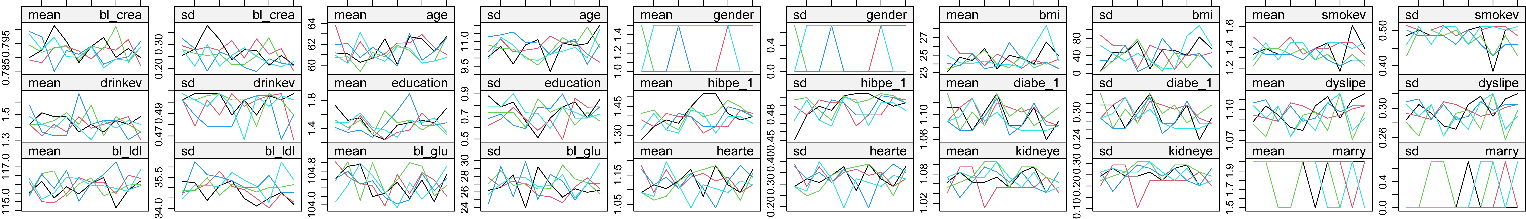


2011cohort


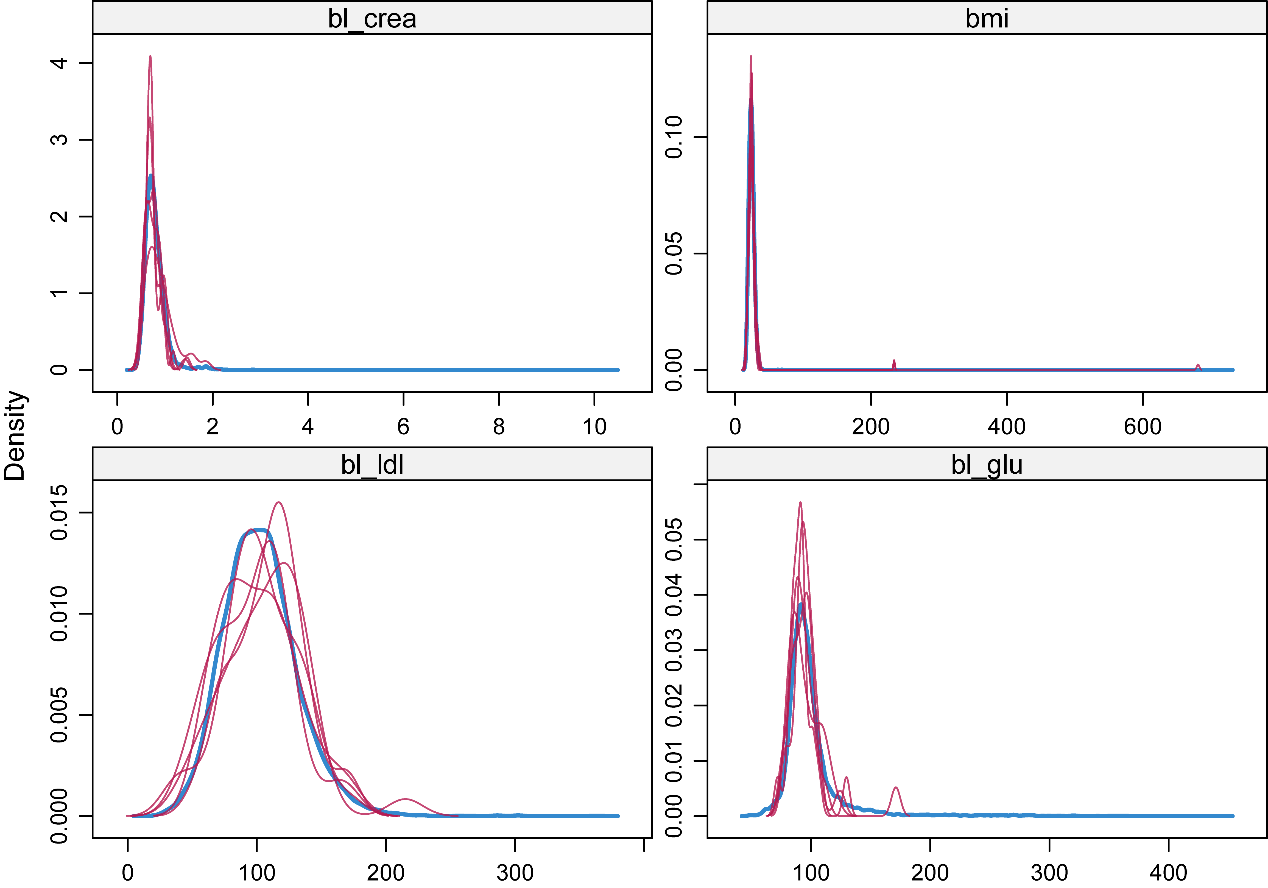


prospective cohort (2015-2020)


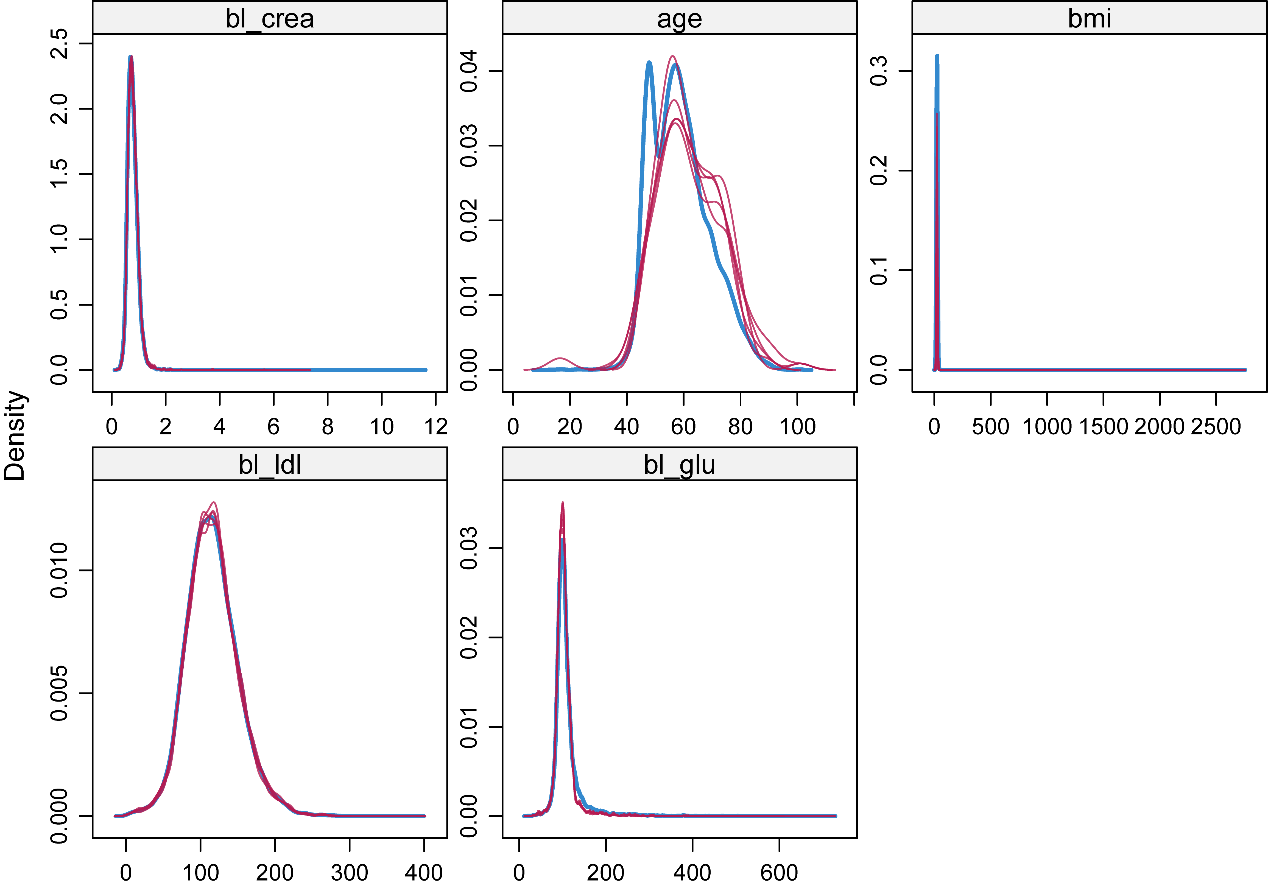
2011 cohort
