# Supplementary material for: Association of estimated glomerular filtration rate with stroke risk in middle-aged and older Chinese adults: an integrated analysis of national and hospital cohorts
Source: Environ Health Prev Med. 2026 May 19;31:33. doi: 10.1265/ehpm.26-00008 (PMC13222745; doi:10.1265/ehpm.26-00008)
Supplement: Supplementary file 2 — Additional file 2: Table S1: Collinearity statistics for all cohorts. [file ehpm-31-033-s002.docx]

| **Variables** | **GVIF** | **Df** | **GVIF^1/2Df^** |
| --- | --- | --- | --- |
| eGFR | 1.319712 | 1 | 1.148787 |
| Age | 1.595304 | 1 | 1.263053 |
| Gender | 2.744166 | 1 | 1.656552 |
| Residence | 1.080618 | 1 | 1.039528 |
| Education level | 1.238630 | 2 | 1.054959 |
| Marital status | 1.141503 | 1 | 1.068412 |
| Smoking history | 2.310084 | 1 | 1.519896 |
| Drinking history | 1.364106 | 1 | 1.167950 |
| BMI | 1.181885 | 1 | 1.087145 |
| LDL-c | 1.047922 | 1 | 1.023681 |
| Kidney disease | 1.032745 | 1 | 1.016240 |
| Diabetes | 1.462061 | 1 | 1.209157 |
| Heart disease | 1.139219 | 1 | 1.067342 |
| Dyslipidemia | 1.174252 | 1 | 1.083629 |
| FBG | 1.389926 | 1 | 1.178951 |
| Hypertension | 1.124171 | 1 | 1.060269 |

**Table S1.** Collinearity Statistics (prospective cohort)

| Collinearity Statistics (2011 cohort) |  |  |  |
| --- | --- | --- | --- |
| **Variables** | **GVIF** | **Df** | **GVIF^1/2Df^** |
| eGFR | 1.413338 | 1 | 1.188839 |
| Age | 1.681268 | 1 | 1.296637 |
| Gender | 2.152000 | 1 | 1.466970 |
| Residence | 1.096449 | 1 | 1.047115 |
| Education level | 1.212805 | 2 | 1.049416 |
| Marital status | 1.152221 | 1 | 1.073415 |
| Smoking history | 1.850110 | 1 | 1.360187 |
| Drinking history | 1.454723 | 1 | 1.206119 |
| BMI | 1.248392 | 1 | 1.117315 |
| LDL-c | 1.036780 | 1 | 1.018224 |
| Kidney disease | 1.036997 | 1 | 1.018331 |
| Diabetes | 1.650849 | 1 | 1.284854 |
| Heart disease | 1.110372 | 1 | 1.053742 |
| Dyslipidemia | 1.209482 | 1 | 1.099764 |
| FBG | 1.618375 | 1 | 1.272154 |
| Hypertension | 1.109884 | 1 | 1.053510 |

Collinearity Statistics (hospital cohort)

| **Variables** | **GVIF** | **Df** | **GVIF^1/2Df^** |
| --- | --- | --- | --- |
| eGFR | 1.168539 | 1 | 1.080990 |
| Age | 1.489969 | 1 | 1.220643 |
| Gender | 1.189513 | 1 | 1.090648 |
| Residence | 1.047063 | 1 | 1.023261 |
| Smoking history | 1.286556 | 1 | 1.134265 |
| Drinking history | 1.137222 | 1 | 1.066406 |
| LDL-c | 1.089859 | 1 | 1.043963 |
| Kidney disease | 1.062558 | 1 | 1.030805 |
| Diabetes | 1.461521 | 1 | 1.208934 |
| Heart disease | 1.357898 | 1 | 1.165289 |
| Dyslipidemia | 1.116526 | 1 | 1.056658 |
| FBG | 1.260696 | 1 | 1.122807 |
| Hypertension | 1.085242 | 1 | 1.041749 |

BMI, body mass index; FBG, fasting blood glucose; LDL-c, low-density lipoprotein cholesterol; eGFR, estimated glomerular filtration rate.
