# Supplementary material for: Association of estimated glomerular filtration rate with stroke risk in middle-aged and older Chinese adults: an integrated analysis of national and hospital cohorts
Source: Environ Health Prev Med. 2026 May 19;31:33. doi: 10.1265/ehpm.26-00008 (PMC13222745; doi:10.1265/ehpm.26-00008)
Supplement: Supplementary file 3 — Additional file 3: Table S2: Baseline characteristics by eGFR category (CHARLS 2011 wave). [file ehpm-31-033-s003.docx]

| **Table S2: Baseline characteristics of individuals classified by categories of the eGFR (2011 wave)** | | | | | | | | |
| --- | --- | --- | --- | --- | --- | --- | --- | --- |
| **Characteristics** | **Overall** | **Categories of eGFR** | | | | | | |
|  |  | **G1** | **G2** | **G3a** | **G3b** | **G4** | **G5** | **P value** |
| **n** | 8467 | 503 | 6924 | 861 | 153 | 21 | 5 |  |
| **Gender (Male) (%)** | 3926 (46.4) | 222 (44.1) | 3196 (46.2) | 420 (48.8) | 75 (49.0) | 9 (42.9) | 4 (80.0) | 0.310 |
| **age (mean (SD)), years** | 59.6 (9.4) | 50.6 (5.5) | 59.0 (8.6) | 67.4 (9.4) | 71.2 (9.4) | 70.0 (11.7) | 63.4 (11.1) | <0.001 |
| **Marriage (Married) (%)** | 7387 (87.2) | 478 (95.0) | 6098 (88.1) | 674 (78.3) | 117 (76.5) | 15 (71.4) | 5 (100.0) | <0.001 |
| **Residence (Rural) (%)** | 5453 (64.4) | 346 (68.8) | 4525 (65.4) | 483 (56.1) | 87 (56.9) | 9 (42.9) | 3 (60.0) | <0.001 |
| **education (%)** |  |  |  |  |  |  |  | <0.001 |
| **Primary school or lower** | 5978 (70.6) | 280 (55.7) | 4883 (70.6) | 677 (78.8) | 119 (77.8) | 15 (71.4) | 4 (80.0) |  |
| **Middle school** | 1649 (19.5) | 147 (29.2) | 1358 (19.6) | 119 (13.9) | 19 (12.4) | 5 (23.8) | 1 (20.0) |  |
| **High school or above** | 835 (9.9) | 76 (15.1) | 680 (9.8) | 63 (7.3) | 15 (9.8) | 1 (4.8) | 0 (0.0) |  |
| **BMI (mean (SD)), kg/m^2^** | 23.5 (3.9) | 23.7 (3.6) | 23.5 (3.9) | 23.5 (4.2) | 23.3 (5.0) | 23.7 (4.2) | 23.7 (3.8) | 0.947 |
| **Drinking (Yes) (%)** | 3319 (39.2) | 201 (40.0) | 2733 (39.5) | 326 (37.9) | 47 (30.7) | 9 (42.9) | 3 (60.0) | 0.250 |
| **Smoking (Yes) (%)** | 3307 (39.1) | 183 (36.4) | 2677 (38.7) | 374 (43.4) | 62 (40.5) | 8 (38.1) | 3 (60.0) | 0.076 |
| **Kidney disease (Yes) (%)** | 500 (5.9) | 27 (5.4) | 377 (5.5) | 69 (8.1) | 16 (10.5) | 6 (28.6) | 5 (100.0) | <0.001 |
| **Diabetes (Yes) (%)** | 1355 (16.1) | 80 (16.2) | 1048 (15.3) | 175 (20.4) | 41 (26.8) | 10 (47.6) | 1 (20.0) | <0.001 |
| **Hypertension (Yes) (%)** | 4079 (48.3) | 165 (33.2) | 3225 (46.7) | 560 (65.0) | 108 (71.1) | 17 (81.0) | 4 (80.0) | <0.001 |
| **Heart disease (Yes) (%)** | 1016 (12.0) | 37 (7.4) | 778 (11.3) | 166 (19.3) | 29 (19.0) | 6 (28.6) | 0 (0.0) | <0.001 |
| **Dyslipidemia (Yes) (%)** | 848 (10.2) | 42 (8.7) | 687 (10.1) | 93 (11.0) | 20 (13.2) | 5 (23.8) | 1 (20.0) | 0.151 |
| **FBG (mean (SD)), mg/dL** | 110.1 (35.6) | 109.0 (36.8) | 109.3 (32.9) | 115.0 (48.5) | 116.6 (42.3) | 157.3 (103.9) | 105.5 (11.2) | <0.001 |
| **LDL-c (mean (SD)), mg/dL** | 117.7 (35.0) | 106.6 (33.9) | 117.9 (34.4) | 121.6 (37.7) | 122.5 (38.5) | 125.3 (38.4) | 104.5 (44.6) | <0.001 |
| **CREA (mean (SD)), mg/dL** | 0.8 (0.2) | 0.5 (0.1) | 0.8 (0.1) | 1.0 (0.1) | 1.3 (0.2) | 1.8 (0.4) | 5.5 (3.5) | <0.001 |
| **eGFR (mean (SD)), mL/min/1.73 m^2^** | 74.6 (12.1) | 94.1 (5.3) | 76.6 (7.5) | 54.3 (3.9) | 39.8 (3.9) | 25.9 (3.7) | 10.4 (5.0) | <0.001 |
| **Stroke (Yes) (%)** | 218 (2.6) | 7 (1.4) | 162 (2.3) | 34 (3.9) | 12 (7.8) | 2 (9.5) | 1 (20.0) | <0.001 |

BMI, body mass index; FBG, fasting blood glucose; LDL-c, low-density lipoprotein cholesterol; CREA, serum creatinine; eGFR, estimated glomerular filtration rate.
