# Supplementary material for: Association of estimated glomerular filtration rate with stroke risk in middle-aged and older Chinese adults: an integrated analysis of national and hospital cohorts
Source: Environ Health Prev Med. 2026 May 19;31:33. doi: 10.1265/ehpm.26-00008 (PMC13222745; doi:10.1265/ehpm.26-00008)
Supplement: Supplementary file 4 — Additional file 4: Table S3: Baseline characteristics by eGFR category (hospital cohort). [file ehpm-31-033-s004.docx]

| **Table S3: Baseline characteristics of individuals classified by categories of the eGFR (hospital cohort)** | | | | | | | | |
| --- | --- | --- | --- | --- | --- | --- | --- | --- |
| **Characteristics** | **Overall** | **Categories of eGFR** | | | | | | |
|  |  | **G1** | **G2** | **G3a** | **G3b** | **G4** | **G5** | **P value** |
| **n** | 1649 | 14 | 826 | 381 | 275 | 90 | 63 |  |
| **Gender (Male) (%)** | 837 (50.8) | 5 (35.7) | 437 (52.9) | 200 (52.5) | 115 (41.8) | 49 (54.4) | 31 (49.2) | 0.027 |
| **age (mean (SD)), years** | 66.4 (11.7) | 50.4 (3.8) | 62.1 (10.7) | 70.0 (10.6) | 72.8 (10.4) | 71.2 (11.4) | 70.0 (12.0) | <0.001 |
| **Marriage (Married) (%)** | 1629 (98.8) | 14 (100.0) | 824 (99.8) | 373 (97.9) | 268 (97.5) | 88 (97.8) | 62 (98.4) | 0.015 |
| **Residence (Rural) (%)** | 1110 (67.3) | 9 (64.3) | 613 (74.2) | 233 (61.2) | 172 (62.5) | 50 (55.6) | 33 (52.4) | <0.001 |
| **Drinking (Yes) (%)** | 53 (3.2) | 2 (14.3) | 25 (3.0) | 12 (3.1) | 7 (2.5) | 3 (3.3) | 4 (6.3) | 0.156 |
| **Smoking (Yes) (%)** | 187 (11.3) | 3 (21.4) | 91 (11.0) | 47 (12.3) | 32 (11.6) | 8 (8.9) | 6 (9.5) | 0.754 |
| **Kidney disease (Yes) (%)** | 291 (17.6) | 1 (7.1) | 104 (12.6) | 80 (21.0) | 62 (22.5) | 24 (26.7) | 20 (31.7) | <0.001 |
| **Diabetes (Yes) (%)** | 608 (36.9) | 4 (28.6) | 272 (32.9) | 140 (36.7) | 122 (44.4) | 40 (44.4) | 30 (47.6) | 0.003 |
| **Hypertension (Yes) (%)** | 1079 (65.4) | 10 (71.4) | 472 (57.1) | 251 (65.9) | 221 (80.4) | 71 (78.9) | 54 (85.7) | <0.001 |
| **Heart disease (Yes) (%)** | 624 (37.8) | 3 (21.4) | 272 (32.9) | 171 (44.9) | 120 (43.6) | 42 (46.7) | 16 (25.4) | <0.001 |
| **Dyslipidemia (Yes) (%)** | 707 (42.9) | 7 (50.0) | 404 (48.9) | 156 (40.9) | 99 (36.0) | 27 (30.0) | 14 (22.2) | <0.001 |
| **FBG (mean (SD)), mg/dL** | 144.0 (75.6) | 136.8 (55.8) | 138.6 (77.4) | 145.8 (70.2) | 151.2 (81.0) | 149.4 (81.0) | 149.4 (61.2) | 0.138 |
| **LDL-c (mean (SD)), mg/dL** | 100.5 (38.7) | 108.3 (42.5) | 104.4 (42.5) | 96.7 (38.7) | 92.8 (38.7) | 88.9 (38.7) | 92.8 (38.7) | <0.001 |
| **CREA (mean (SD)), mg/dL** | 1.2 (1.1) | 0.5 (0.1) | 0.8 (0.1) | 1.0 (0.1) | 1.3 (0.2) | 2.1 (0.5) | 5.5 (3.3) | <0.001 |
| **eGFR (mean (SD)), mL/min/1.73 m^2^** | 57.8 (19.7) | 94.2 (2.9) | 73.2 (7.3) | 53.1 (4.0) | 38.3 (4.3) | 23.8 (4.4) | 8.9 (3.7) | <0.001 |
| **Stroke (Yes) (%)** | 839 (50.9) | 3 (21.4) | 287 (34.7) | 235 (61.7) | 187 (68.0) | 72 (80.0) | 55 (87.3) | <0.001 |

**FBG, fasting blood glucose; LDL-c, low-density lipoprotein cholesterol; CREA, serum creatinine; eGFR, estimated glomerular filtration rate.**
