# Supplementary material for: Association of estimated glomerular filtration rate with stroke risk in middle-aged and older Chinese adults: an integrated analysis of national and hospital cohorts
Source: Environ Health Prev Med. 2026 May 19;31:33. doi: 10.1265/ehpm.26-00008 (PMC13222745; doi:10.1265/ehpm.26-00008)
Supplement: Supplementary file 5 — Additional file 5: Table S4: Association between eGFR and stroke (CHARLS 2011 wave). [file ehpm-31-033-s005.docx]

| **Table S4: Association between the eGFR and Stroke (2011 wave).** | | | | | | | | |
| --- | --- | --- | --- | --- | --- | --- | --- | --- |
| **eGFR** | **Categories** | | | | | | **P for trend** | **Continuous**  **Per 1mL/min/1.73 m^2^ decrease** |
|  | **G1** | **G2** | **G3a** | **G3b** | **G4** | **G5** |  |  |
| **Median** | 92.573 | 77.436 | 54.964 | 40.536 | 27.262 | 13.180 | - | - |
| **Cases, n (%)** | 7 (1.4) | 162 (2.3) | 34 (3.9) | 12 (7.8) | 2 (9.5) | 1 (20.0) | - | - |
| **Model 1**  **OR (95% CI)** | ref | 1.698  (0.855–4.016) | 2.913  (1.361–7.211) | 6.030  (2.380–16.473) | 7.459  (1.064–33.427) | 17.714  (0.849–141.120) | <0.001 | 1.034  (1.024–1.044) |
| **Model 2**  **OR (95% CI)** | ref | 1.203  (0.594–2.881) | 1.509  (0.665–3.890) | 2.887  (1.074–8.298) | 3.366  (0.465–15.890) | 9.977  (0.468–82.565) | 0.003 | 1.024  (1.012–1.036) |
| **Model 3**  **OR (95% CI)** | ref | 1.022  (0.499–2.464) | 1.198  (0.520–3.121) | 2.421  (0.890–7.029) | 2.123  (0.285–10.486) | 7.091  (0.319–64.336) | 0.016 | 1.020  (1.008–1.032) |

Model 1: adjusted for no variables;

Model 2: adjusted for age, gender, marriage, residence, education, and BMI.

Model 3: adjusted for variables included in Model 2 and drinking history, smoking history, kidney disease, diabetes, hypertension, heart disease, dyslipidemia, FBG, and LDL-c.

eGFR, estimated glomerular filtration rate; OR, odds ratio; CI, confidence interval.
