# Supplementary material for: Association of estimated glomerular filtration rate with stroke risk in middle-aged and older Chinese adults: an integrated analysis of national and hospital cohorts
Source: Environ Health Prev Med. 2026 May 19;31:33. doi: 10.1265/ehpm.26-00008 (PMC13222745; doi:10.1265/ehpm.26-00008)
Supplement: Supplementary file 6 — Additional file 6: Table S5: Association between eGFR and stroke (hospital cohort). [file ehpm-31-033-s006.docx]

| **Table S5: Association between the eGFR and Stroke (hospital cohort).** | | | | | | | | |
| --- | --- | --- | --- | --- | --- | --- | --- | --- |
| **eGFR** | **Categories** | | | | | | **P for trend** | **Continuous**  **Per 1mL/min/1.73 m^2^ decrease** |
|  | **G1** | **G2** | **G3a** | **G3b** | **G4** | **G5** |  |  |
| **Median** | 92.873 | 73.235 | 53.307 | 39.299 | 24.831 | 9.111 | - | - |
| **Cases, n (%)** | 3 (21.4) | 287 (34.7) | 235 (61.7) | 187 (68.0) | 72 (80.0) | 55 (87.3) | - | - |
| **Model 1**  **OR (95% CI)** | ref | 1.952  (0.604–8.683) | 5.902  (1.808–26.414) | 7.792  (2.364–35.087) | 14.667  (4.097–70.050) | 25.208  (6.389–131.418) | <0.001 | 1.045  (1.039–1.051) |
| **Model 2**  **OR (95% CI)** | ref | 0.879  (0.262–4.018) | 1.619  (0.473–7.482) | 1.835  (0.528–8.567) | 3.970  (1.055–19.641) | 7.847  (1.895–42.380) | <0.001 | 1.029  (1.022–1.035) |
| **Model 3**  **OR (95% CI)** | ref | 1.549  (0.404–8.310) | 3.027  (0.774–16.450) | 2.739  (0.690–15.231) | 6.646  (1.546–38.666) | 10.582  (2.213–67.610) | <0.001 | 1.026  (1.019–1.033) |

Model 1: adjusted for no variables;

Model 2: adjusted for age, gender, marriage, and residence.

Model 3: adjusted for variables included in Model 2 and drinking history, smoking history, kidney disease, diabetes, hypertension, heart disease, dyslipidemia, FBG, and LDL-c.

eGFR, estimated glomerular filtration rate; OR, odds ratio; CI, confidence interval.
