# Supplementary material for: Association of estimated glomerular filtration rate with stroke risk in middle-aged and older Chinese adults: an integrated analysis of national and hospital cohorts
Source: Environ Health Prev Med. 2026 May 19;31:33. doi: 10.1265/ehpm.26-00008 (PMC13222745; doi:10.1265/ehpm.26-00008)
Supplement: Supplementary file 7 — Additional file 7: Table S6: Association between eGFR and ischemic stroke in the hospital cohort. [file ehpm-31-033-s007.docx]

| **Table S6: Association between eGFR and ischemic stroke in the hospital cohort.** | | | | | | | | |
| --- | --- | --- | --- | --- | --- | --- | --- | --- |
| **eGFR** | **Categories** | | | | | | **P for trend** | **Continuous**  **Per 1mL/min/1.73 m^2^ decrease** |
|  | **G1** | **G2** | **G3a** | **G3b** | **G4** | **G5** |  |  |
| **Median** | 93.158 | 73.969 | 53.496 | 38.489 | 22.203 | 9.611 | - | - |
| **Cases, n (%)** | 1 (8.3) | 120 (18.2) | 92 (38.7) | 67 (43.2) | 27 (60.0) | 30 (78.9) | - | - |
| **Model 1**  **OR (95% CI)** | ref | 2.449  (0.470–44.980) | 6.932  (1.316–127.756) | 8.375  (1.571–154.974) | 16.500  (2.841–315.053) | 41.250  (6.621–814.161) | <0.001 | 1.042  (1.034–1.049) |
| **Model 2**  **OR (95% CI)** | ref | 1.268  (0.231–23.702) | 2.123  (0.378–40.032) | 1.888  (0.327–35.939) | 4.433  (0.707–87.142) | 13.125  (1.981–265.495) | <0.001 | 1.026  (1.017–1.034) |
| **Model 3**  **OR (95% CI)** | ref | 3.642  (0.488–83.579) | 6.794  (0.887–157.699) | 5.302  (0.678–124.152) | 13.630  (1.599–332.115) | 26.368  (2.939–661.221) | <0.001 | 1.025  (1.016–1.034) |

Model 1: adjusted for no variables;

Model 2: adjusted for age, gender, marriage, and residence.

Model 3: adjusted for variables included in Model 2 and drinking history, smoking history, kidney disease, diabetes, hypertension, heart disease, dyslipidemia, FBG, and LDL-c.

eGFR, estimated glomerular filtration rate; OR, odds ratio; CI, confidence interval.
