# Supplementary material for: Association of estimated glomerular filtration rate with stroke risk in middle-aged and older Chinese adults: an integrated analysis of national and hospital cohorts
Source: Environ Health Prev Med. 2026 May 19;31:33. doi: 10.1265/ehpm.26-00008 (PMC13222745; doi:10.1265/ehpm.26-00008)
Supplement: Supplementary file 8 — Additional file 8: Table S7: Association between eGFR and hemorrhagic stroke in the hospital cohort. [file ehpm-31-033-s008.docx]

| **Table S7: Association between eGFR and hemorrhagic stroke in the hospital cohort.** | | | | | | | | |
| --- | --- | --- | --- | --- | --- | --- | --- | --- |
| **eGFR** | **Categories** | | | | | | **P for trend** | **Continuous**  **Per 1mL/min/1.73 m^2^ decrease** |
|  | **G1** | **G2** | **G3a** | **G3b** | **G4** | **G5** |  |  |
| **Median** | 94.236 | 75.515 | 53.975 | 39.398 | 25.220 | 8.548 | - | - |
| **Cases, n (%)** | 2 (15.4) | 167 (23.7) | 143 (49.5) | 120 (57.7) | 45 (71.4) | 25 (75.8) | - | - |
| **Model 1**  **OR (95% CI)** | ref | 1.704  (0.452–11.084) | 5.387  (1.415–35.211) | 7.500  (1.952–49.258) | 13.750  (3.288–94.808) | 17.188  (3.680–127.848) | <0.001 | 1.045  (1.038–1.053) |
| **Model 2**  **OR (95% CI)** | ref | 0.792  (0.205–5.223) | 1.525  (0.385–10.172) | 1.928  (0.480–12.957) | 3.761  (0.862–26.544) | 5.660  (1.158–43.214) | <0.001 | 1.029  (1.021–1.037) |
| **Model 3**  **OR (95% CI)** | ref | 1.114  (0.272–7.547) | 2.132  (0.509–14.647) | 2.264  (0.532–15.679) | 4.846  (1.042–35.436) | 5.840  (1.086–47.402) | <0.001 | 1.027  (1.018–1.035) |

Model 1: adjusted for no variables;

Model 2: adjusted for age, gender, marriage, and residence.

Model 3: adjusted for variables included in Model 2 and drinking history, smoking history, kidney disease, diabetes, hypertension, heart disease, dyslipidemia, FBG, and LDL-c.

eGFR, estimated glomerular filtration rate; OR, odds ratio; CI, confidence interval.
