# Supplementary material for: Association of estimated glomerular filtration rate with stroke risk in middle-aged and older Chinese adults: an integrated analysis of national and hospital cohorts
Source: Environ Health Prev Med. 2026 May 19;31:33. doi: 10.1265/ehpm.26-00008 (PMC13222745; doi:10.1265/ehpm.26-00008)
Supplement: Supplementary file 9 — Additional file 9: Table S8: Association between eGFR and stroke after multiple imputation (prospective cohort). [file ehpm-31-033-s009.docx]

| **Table S8: Association between the eGFR and Stroke (2015-2020, multiple imputation).** | | | | | | |
| --- | --- | --- | --- | --- | --- | --- |
| **eGFR** | **Categories** | | | | **P for trend** | **Continuous**  **Per 1mL/min/1.73 m2 decrease** |
|  | **Quartile 1** | **Quartile 2** | **Quartile 3** | **Quartile 4** |  |  |
| **Median** | 59.82 | 72.47 | 78.75 | 85.29 |  |  |
| **Model 1**  **HR (95% CI)** | 2.660  (2.078–3.404) | 1.862  (1.435–2.415) | 1.537  (1.173–2.013) | Ref | <0.001 | 1.020 (1.015–1.026) |
| **Model 2**  **HR (95% CI)** | 2.075  (1.554–2.771) | 1.500  (1.123–2.003) | 1.364  (1.032–1.802) | Ref | <0.001 | 1.014 (1.007–1.020) |
| **Model 3**  **HR (95% CI)** | 1.988  (1.487–2.659) | 1.476  (1.102–1.978) | 1.335  (1.008–1.767) | Ref | <0.001 | 1.012 (1.006–1.019) |
| Model 1: adjusted for no variables; | | | | | | |
| Model 2: adjusted for age, gender, marriage, residence, education, and BMI. | | | | | | |
| Model 3: adjusted for variables included in Model 2 and drinking history, smoking history, kidney disease, diabetes, hypertension, heart disease, dyslipidemia, FBG, and LDL-c. | | | | | | |
| eGFR, estimated glomerular filtration rate; HR, hazard ratio; CI, confidence interval. | | | | | | |
