# Supplementary material for: Association of estimated glomerular filtration rate with stroke risk in middle-aged and older Chinese adults: an integrated analysis of national and hospital cohorts
Source: Environ Health Prev Med. 2026 May 19;31:33. doi: 10.1265/ehpm.26-00008 (PMC13222745; doi:10.1265/ehpm.26-00008)
Supplement: Supplementary file 10 — Additional file 10: Table S9: Association between eGFR and stroke after multiple imputation (CHARLS 2011 wave). [file ehpm-31-033-s010.docx]

| **Table S9: Association between the eGFR and Stroke in 2011 wave (multiple imputation)** | | | | | | | | |
| --- | --- | --- | --- | --- | --- | --- | --- | --- |
| **eGFR** | **Categories** | | | | | | **P for trend** | **Continuous**  **Per 1mL/min/1.73 m^2^ decrease** |
|  | **G1** | **G2** | **G3a** | **G3b** | **G4** | **G5** |  |  |
| **Median** | 92.847 | 77.722 | 54.761 | 40.634 | 26.481 | 8.143 | - | - |
| **Model 1**  **OR (95% CI)** | ref | 2.048  (1.095–3.830) | 4.813  (2.493–9.292) | 8.591  (3.692–19.992) | 7.009  (1.295–37.943) | 11.709  (1.086–126.260) | <0.001 | 1.040  (1.032–1.047) |
| **Model 2**  **OR (95% CI)** | ref | 1.367  (0.714–2.616) | 2.252  (1.097–4.624) | 3.438  (1.345–8.789) | 2.774  (0.487–15.797) | 5.093  (0.409–63.385) | <0.001 | 1.027  (1.018–1.036) |
| **Model 3**  **OR (95% CI)** | ref | 1.213  (0.640–2.299) | 1.791  (0.894–3.591) | 2.637  (1.074–6.475) | 1.666  (0.300–99.251) | 3.731  (0.313–44.545) | <0.001 | 1.021  (1.012–1.030) |

Model 1: adjusted for no variables;

Model 2: adjusted for age, gender, marriage, residence, education, and BMI.

Model 3: adjusted for variables included in Model 2 and drinking history, smoking history, kidney disease, diabetes, hypertension, heart disease, dyslipidemia, FBG, and LDL-c.

eGFR, estimated glomerular filtration rate; OR, odds ratio; CI, confidence interval.
