# Supplementary figures and images for: Association of estimated glomerular filtration rate with stroke risk in middle-aged and older Chinese adults: an integrated analysis of national and hospital cohorts
Source: Environ Health Prev Med. 2026 May 19;31:33. doi: 10.1265/ehpm.26-00008 (PMC13222745; doi:10.1265/ehpm.26-00008)

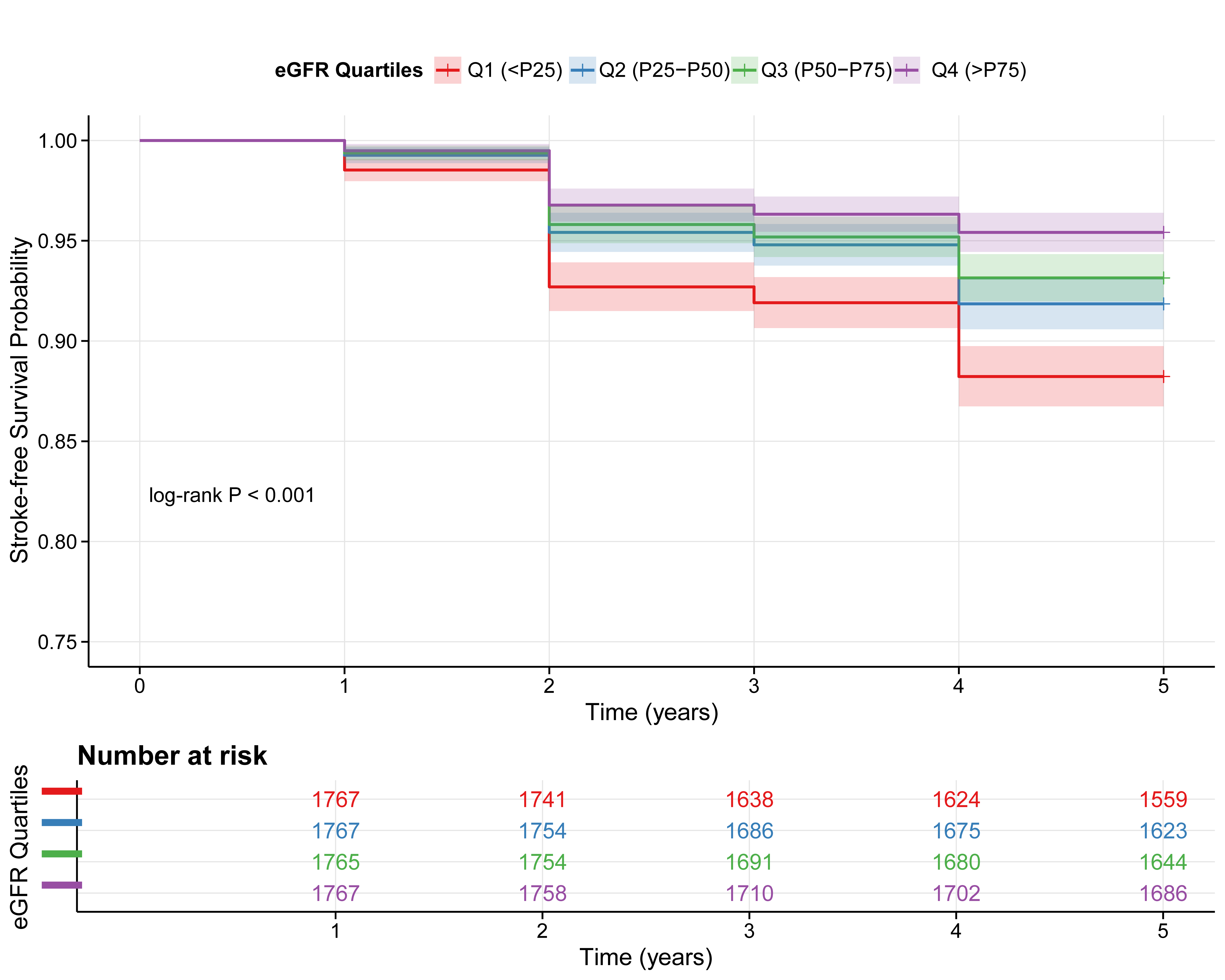

Supplement: Supplementary file 11 — Additional file 11: Figure S1: Kaplan-Meier survival curve for new-onset stroke of CHARLS participants by eGFR quartile. [file ehpm-31-033-s011.tif]

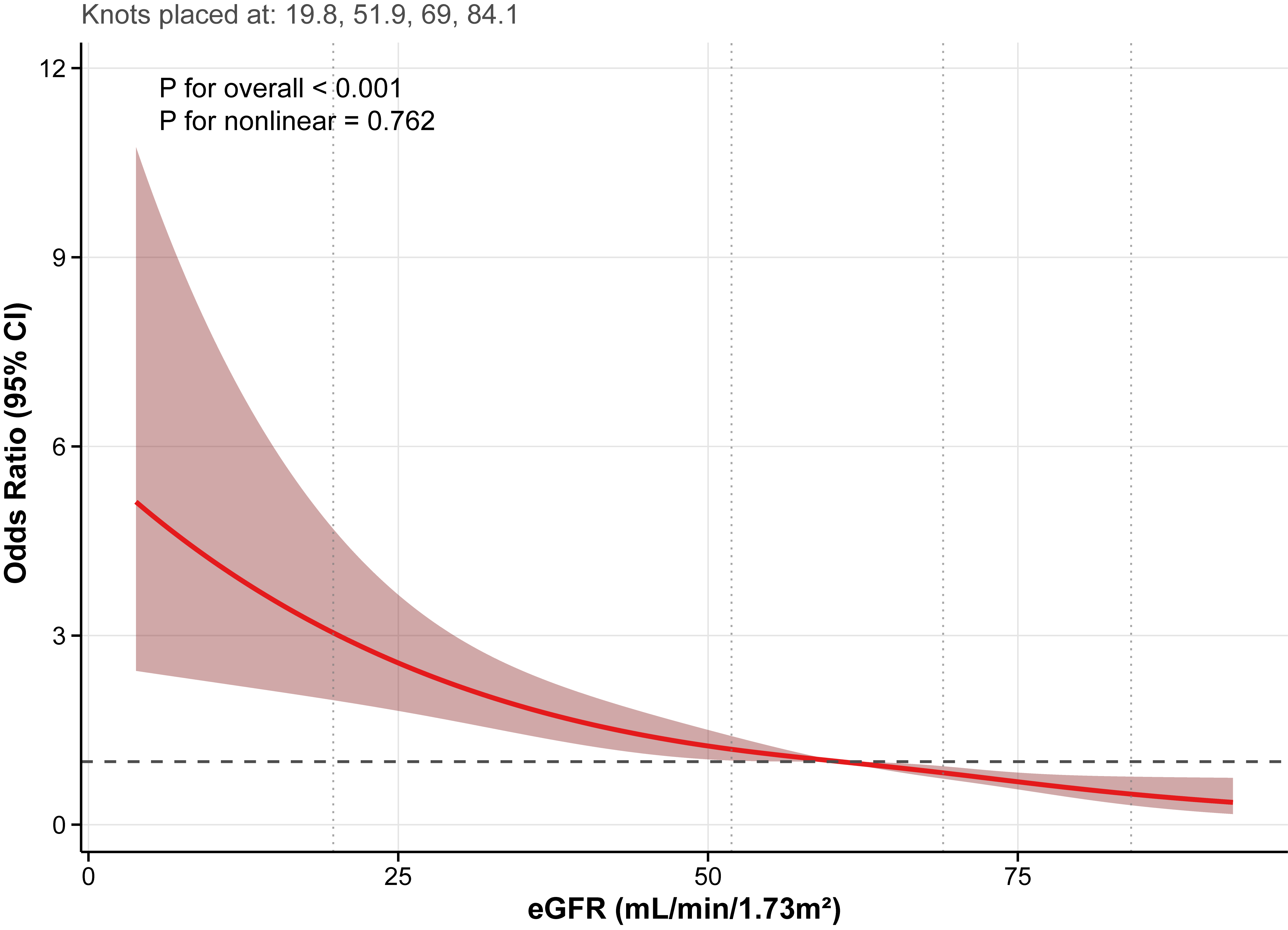

Supplement: Supplementary file 12 — Additional file 12: Figure S2: Dose-response relationship between eGFR and stroke (hospital cohort, RCS analysis). [file ehpm-31-033-s012.tif]

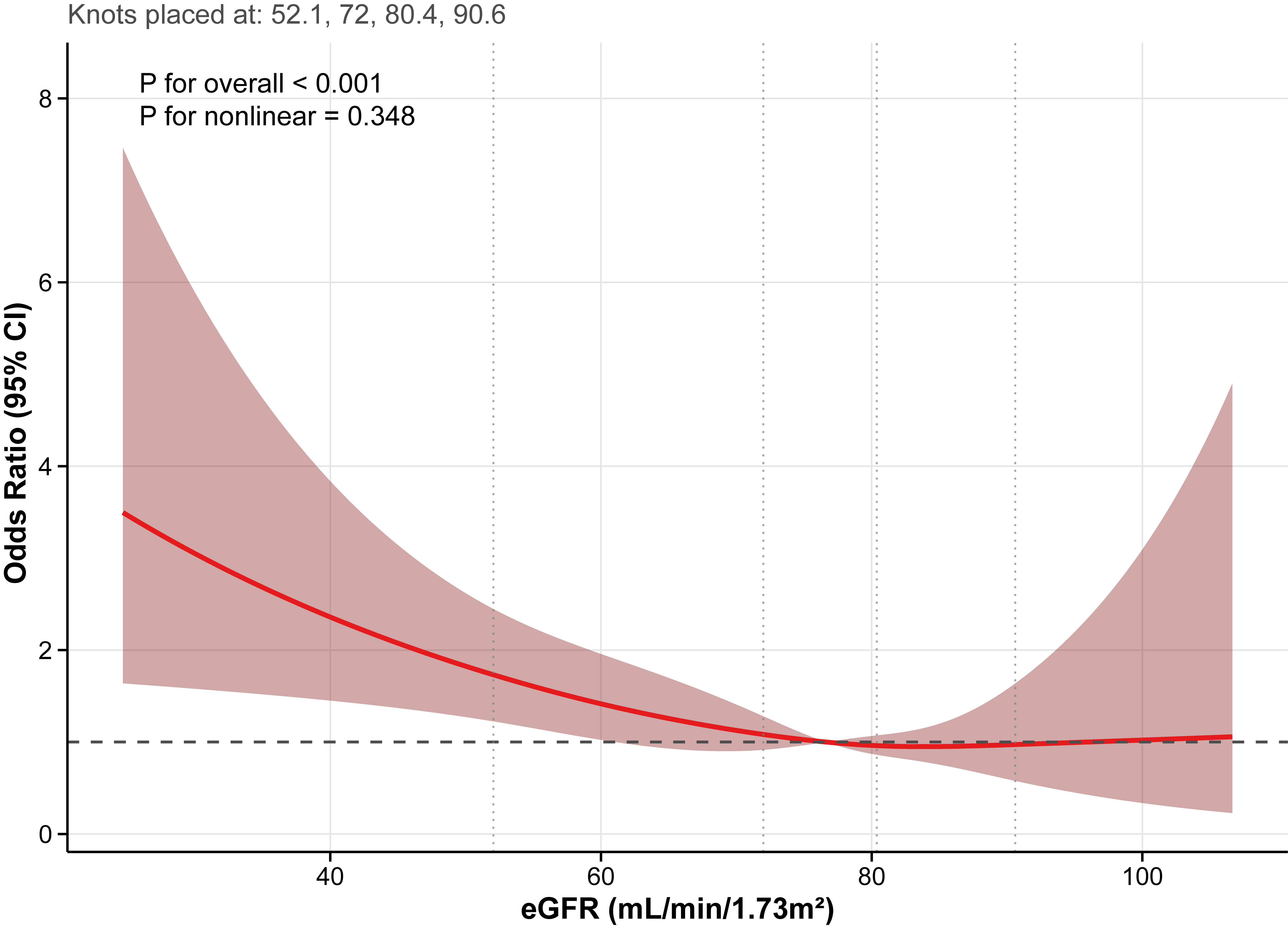

Supplement: Supplementary file 13 — Additional file 13: Figure S3: Dose-response relationship between eGFR and stroke (CHARLS 2011 wave, RCS analysis). [file ehpm-31-033-s013.tif]

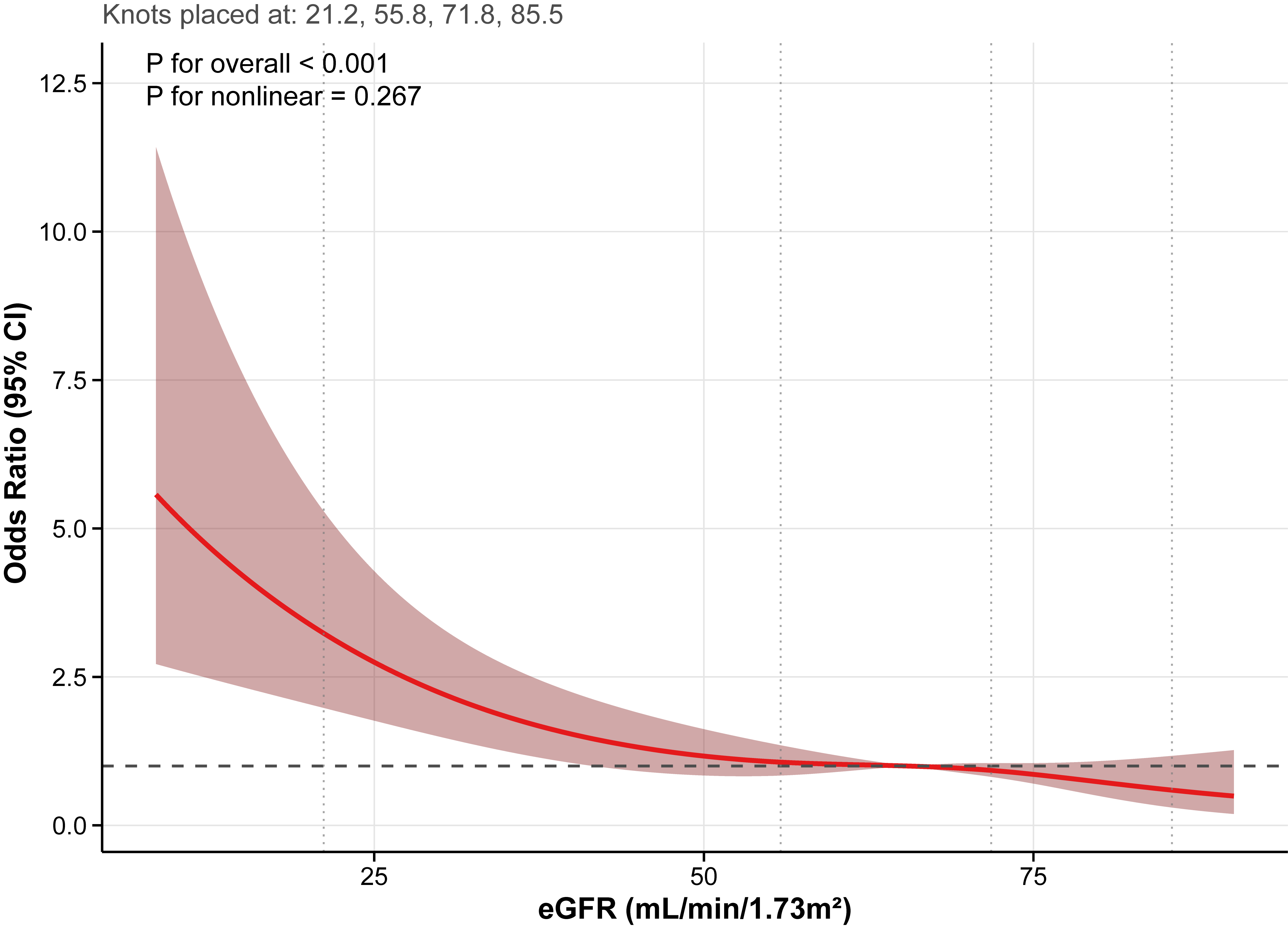

Supplement: Supplementary file 14 — Additional file 14: Figure S4: Dose-response relationship between eGFR and ischemic stroke in the hospital cohort (RCS analysis). [file ehpm-31-033-s014.tif]

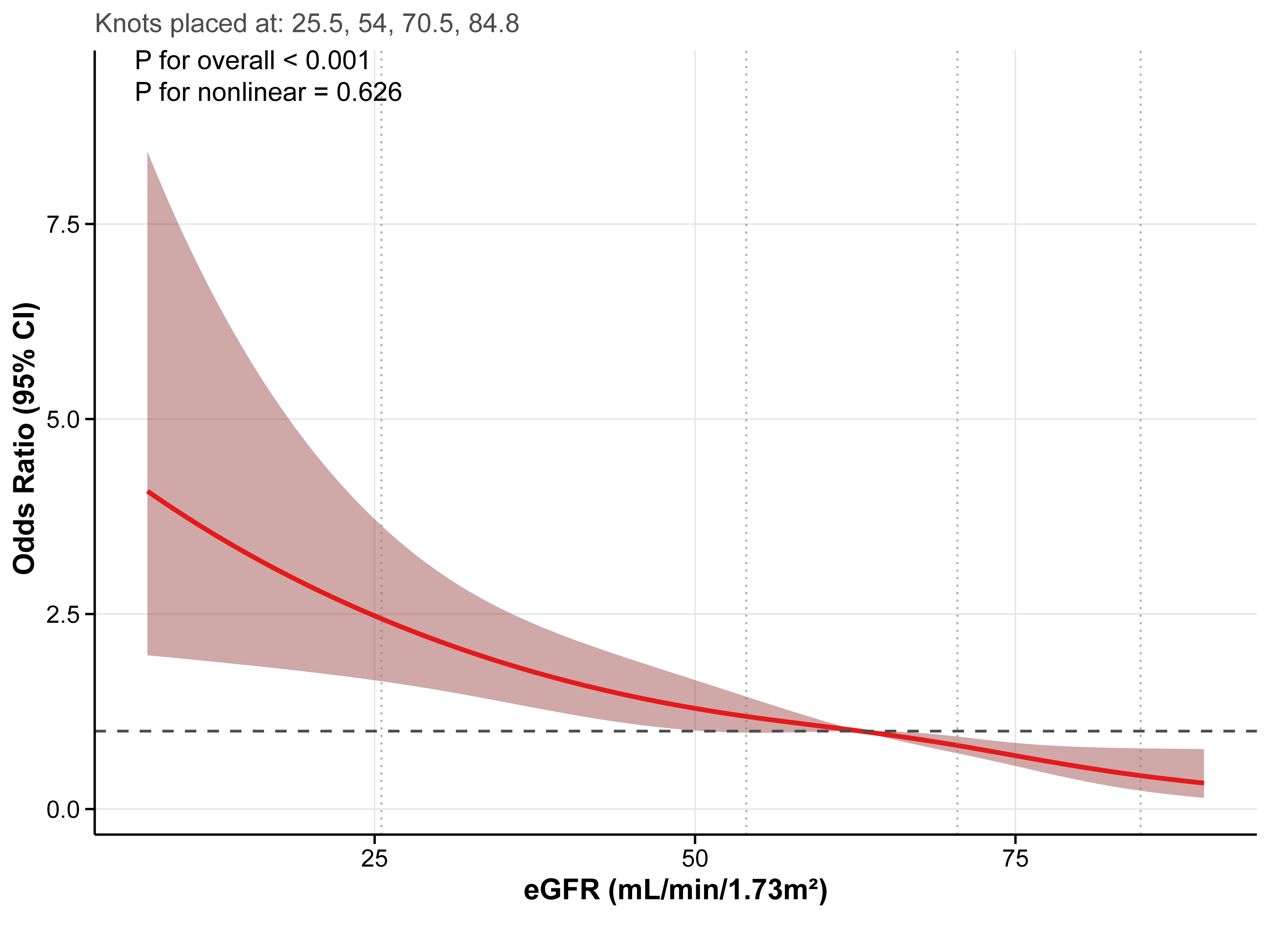

Supplement: Supplementary file 15 — Additional file 15: Figure S5: Dose-response relationship between eGFR and hemorrhagic stroke in the hospital cohort (RCS analysis). [file ehpm-31-033-s015.tif]

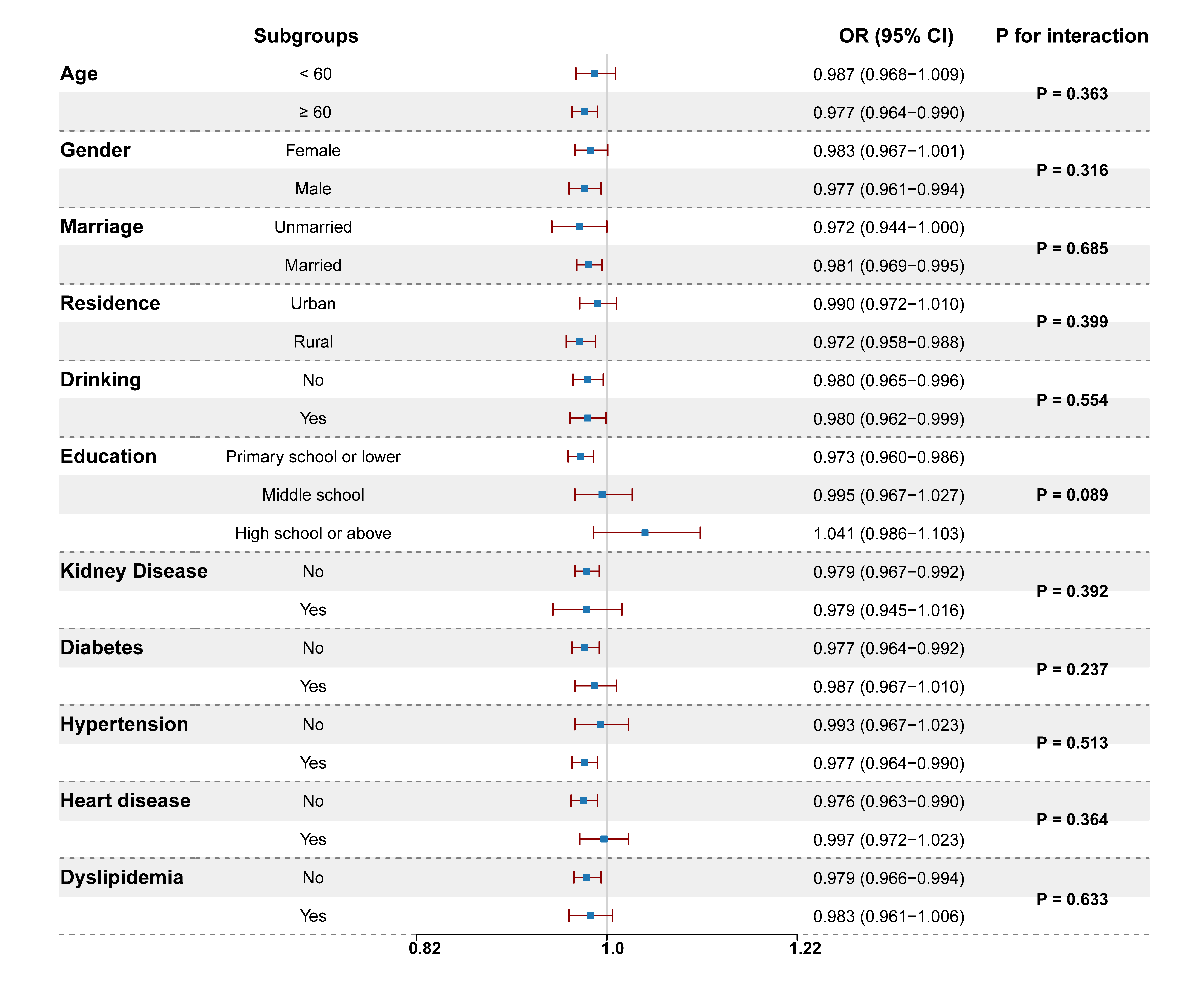

Supplement: Supplementary file 16 — Additional file 16: Figure S6: Subgroup analysis of the eGFR-stroke association (CHARLS 2011 wave). [file ehpm-31-033-s016.tif]
